# Intercropping enhances microbial community diversity and ecosystem functioning in maize fields

**DOI:** 10.3389/fmicb.2022.1084452

**Published:** 2023-01-04

**Authors:** Xiwen Xiao, Lei Han, Hongri Chen, Jianjun Wang, Yuping Zhang, Ang Hu

**Affiliations:** ^1^College of Resources and Environment, Hunan Agricultural University, Changsha, China; ^2^State Key Laboratory of Lake Science and Environment, Nanjing Institute of Geography and Limnology, Chinese Academic of Sciences, Nanjing, China

**Keywords:** intercropping, soil properties, bacterial and fungal diversity, ecosystem functions, effect size (effect magnitude)

## Abstract

**Background and aims:**

Intercropping, a widely used planting pattern, could affect soil physicochemical properties, microbial community diversity, and further crop yields. However, its impacts on soil microbial diversity and ecosystem functioning and further soil sustainability are poorly understood.

**Methods:**

We conducted field experiments by intercropping maize with four important crops (i.e., sesame, peanut, soybean, and sweet potato), and examined soil microbial community diversity and ecosystem functioning such as microbial biomass and enzyme activities under monocropping and intercropping. We quantified their intercropping effects on microbial diversity and ecosystem functions with effect size metric Cohen d by comparing to the monocropping of maize.

**Results:**

We found that the four intercropping systems significantly increased soil aggregates in respective of the 2–0.25 mm grain size. Intercropping consistently elevated ecosystem functioning, such as soil enzyme activities of urease, phosphatase, and catalase, soil microbial biomass carbon and soil microbial biomass nitrogen. The Cohen d of bacterial richness also increased from 0.39 to 2.36, the latter of which was significant for maize/peanut intercropping. Notably, these ecosystem functions were strongly associated with the diversity of bacteria and fungi and the relative abundance of their ecological clusters identified with network analysis.

**Conclusion:**

Together, our findings indicate that intercropping generally affected soil physicochemical properties, ecosystem functions, and promoted microbial community diversity. More importantly, our findings highlight the important roles of microbial diversity of ecological clusters (that is, network modules) in maintaining ecosystem functioning after intercropping. These results will help to better understand the microbial diversity and ecosystem function in intercropping systems and guide agricultural practice.

## Introduction

Intercropping systems, also known as polyculture or mixed cropping, are the growing of different species of plants on the same field during specific periods (Xu et al., 2020). It is a traditional model of farming that can be used to reduce relative inputs to achieve sustainable intensification, and improve the quality of agriculture by taking advantage of complementary species relationships (Xu et al., 2020). Intercropping can provide greater ecosystem services, such as those related to climate change mitigation and ecosystem restoration (Pierre et al., 2022). Intercropping is generally more productive than monocropping because it could protect soil fertility by better using resources, suppressing runoff and soil erosion, retaining water and nutrients, and reducing weed and pest infestation (Wang et al., 2014; Nyawade et al., 2019a). Compared to monocropping, intercropped soils have greater microbial biomass and respiration (Chen et al., 2019), which may be due to the fact that intercropping can increase biomass as well as litter, and promote nitrogen production (Cong et al., 2015). In addition, crop species differ in their ability to release, capture, or retain specific soil nutrients. If crops in an intercropping system could balance the ability to obtain different nutrients, the soil can benefit from multiple elements over time (Güldner and Krausmann, 2017; Choudhary and Choudhury, 2018). However, few studies simultaneously consider both microbial diversity and ecosystem functions affected by intercropping, and further, how these two aspects are linked.

Generally, intercropping could affect microbial diversity and ecosystem functions *via* its effect in soil properties (Nyawade et al., 2019b; Curtright and Tiemann, 2021). Intercropping can affect the content and distribution of soil aggregates to improve soil physicochemical properties (Hu et al., 2022). For instance, soil macro-and microaggregates are higher in maize/faba bean or maize/pigeon pea intercropping systems than in the monocropping system of maize (Garland et al., 2016; Tian et al., 2019), which could further increase soil stability (Tong et al., 2020). Regarding ecosystem functions, intercropping can influence the cycling of key nutrients by increasing soil enzyme activity and microbial biomass (Curtright and Tiemann, 2021). For instance, the invertase, urease, and microbial biomass carbon contents are notably higher in the Solanum tuberosum/Malus domestica intercropping systems in terraces (Xiao et al., 2021). Dehydrogenase activity and microbial biomass carbon in smallholder farms could be elevated by potato/legume intercropping systems (Nyawade et al., 2019b). Microbial community diversity is also highly relevant to cropping systems (Yang et al., 2016). For instance, tree-based intercropping system increases arbuscular mycorrhizal fungal richness and contains several taxa not present in the conventional monocropping system (Bainard et al., 2012). Tobacco/peanut intercropping systems could affect soil bacterial community structure by increasing the proportions of Bacillus and Lactococcus (Gao et al., 2019). Legume-based intercropping systems can increase symbiotic and non-symbiotic beneficial population to improve rhizobacterial community diversity (Chamkhi et al., 2022). In addition, compared to the intercropping without legumes, legume-based intercropping are more likely to increase microbial diversity (Wang et al., 2020). In general, the diversity of bacteria and fungi affects soil ecosystem processes and functions, and the abundant bacteria and fungi help retain most nutrients and drive nutrient recycling, maintain soil structure, and convert organic carbon (Lange et al., 2014; Sugiyama et al., 2014; Lisuma et al., 2020; Ma et al., 2021). Although intercropping could have positive effects on soil properties, microbial diversity and functions, these influences remain elusive especially regarding the links between diversity and ecosystem functions.

In this study, we performed field experiments with the monocropping of maize and its intercropping with another four combinations, that is, maize/sesame, maize/peanut, maize/soybean, and maize/sweet potato. We further examined soil physicochemical properties (e.g., aggregates and nutrients), ecosystem functions (e.g., microbial biomass and enzyme activities), and the diversity of bacteria and fungi in these monocropping and intercropping systems. We hypothesized that, compared to monocropping, intercropping could (1) improve soil physicochemical properties and ecosystem functions, (2) increase microbial diversity, and (3) influence the association between microbial diversity and ecosystem functions.

## Materials and methods

### Site description and experimental design

During 2013–2017, we performed 4-year field experiments at the Yonghe experimental site, located in Yanxi Town, Liuyang, Hunan, China (113°49′15″E, 28°19′41″N; 98 m a.s.l.). The soil texture was river tide soil with pH of 5.69, and the total organic carbon, total nitrogen, phosphorus, and potassium of 11.30, 1.56, 0.48, and 14.48 g/kg, respectively. The available nitrogen, phosphorus, and potassium were 59.67, 122.3, and 152.24 mg/kg, respectively. There were five spatial management treatments in a randomized complete block design with three replicates of each treatment. The five treatments T1–T5 included: T1, 10 rows of maize; T2, 6 rows of maize × 4 rows of sesame; T3, 6 rows of maize × 4 rows of peanut; T4, 6 rows of maize × 4 rows of soybean; T5, 10 rows of maize × 9 rows of sweet potato.

**Figure 1:**
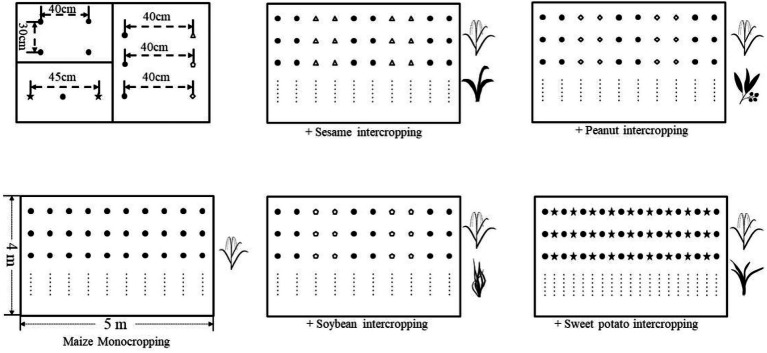
Schematic representation of field experimental setups in this study. The cropping patterns were consisted of the monocropping of maize and its four intercropping systems with sesame, peanut, soybean, and sweet potato.

The row spacing and plant spacing were 40 and 30 cm, respectively, in both monocropping and intercropping, except for maize/sweet potato with a row spacing of 45 cm (Figure 1). The crop cultivar used was Yedan 13 for maize, Xiangzhi 2 for sesame, Xianghua 120 for peanut, Jiunong 22 for soybean, and Shixiu 1 for sweet potato.

Experimental plot was 5 × 4 m in width and length and with an area of 20 m^2^. At the time of sowing three seeds, maize was placed in each hole, and only one seedling was retained in each hole at the three-leaf stage. For fertilizer application, we applied approximately 240 kg ha^−1^ N, 150 kg ha^−1^ P, and 150 kg ha^−1^ K in all treatments during planting. All crops were planted and fertilized at the same time except for sweet potato. Sweet potato was planted after corn, and thus no additional fertilizer was additionally applied. Specifically, fused calcium magnesium phosphate was applied as basal fertilizers, potassium chloride as the basal (50%), and earring (50%) fertilizers, and urea as the basal (40%), seedling (30%), and earring (30%) fertilizers.

### Sampling procedures and analysis

In 2017, soil samples were collected from surface soil depth (0–20 cm) of each treatment at the peak of maize growth stage. The composite samples were air dried and sieved through 1 and 0.25 mm mesh, respectively. We measured soil physiochemical variables. Soil moisture was determined by oven-drying, soil pH with a pH electrode, total organic carbon (TOC) with potassium dichromate oxidation, total nitrogen (TN) with the Kjeldahl method, total phosphorus (TP) with NaOH melting molybdenum antimony anti-colorimetry, total potassium (TK) with NaOH melting flame photometer, soil available nitrogen (AN) with the alkaline hydrolysis diffusion method, soil available phosphorus (AP) with sodium bicarbonate extraction, soil available potassium (AK) with ammonium acetate extraction and flame photometry, and soil aggregates with wet-sieving (Bao, 2000; Du et al., 2015; Zeng et al., 2018; Tian et al., 2019; Liu Z. et al., 2022). Soil microbial biomass carbon (MBC) and microbial biomass nitrogen (MBN) were determined by the chloroform-fumigation extraction method (Vance et al., 1987). Soil enzymes, including urease, phosphatase, and catalase, were extracted and measured as previously described (Johnson and Temple, 1964; Page, 1982; Guan, 1986). Briefly, urease was determined by incubating 10 g of soils with 10 ml of 10% urea solution for 24 h at 37°C. Phosphatase was measured by incubating 1.0 g soils with 4 ml of 5% Na_2_RPO_4_ solution for 24 h at 37°C, and catalase was measured by incubating 5.0 g soils with 5 ml of 0.3% H_2_O_2_ for 30 min at 30°C.

At maize maturity, the grain and straw yield were measured in each plot. We further collected three plant samples from each plot randomly and determined the grain nitrogen (Grain N), grain phosphorus (Grain P), straw nitrogen (Straw N), and straw phosphorus (Straw P). All experiments were performed according to Soil and Agricultural Chemistry Analysis (Bao, 2000).

### Soil DNA sequencing

Frozen-dry soil (0.5 g) was used to extract DNA by the FastDNA SPIN Kit (MP Biomedicals, United States), according to the manufacturer’s instructions. The bacterial 16S rRNA gene was amplified using 515F (5′-GTGCCAGCMGCCGCGGTAA-3′) and 806R (5′-GGACTA CHVGGGTWTCTAAT-3′) targeting the V4 region (Caporaso et al., 2011); the fungal ITS region was amplified using ITS1F (5′-CTTGGTCATTTAGAGGAAGTAA-3′) targeting the ITS1 region (Smith and Peay, 2014). The bacterial and fungal communities were rarefied at 83,000 and 100,000 sequences, respectively. The bacterial 16S rRNA genes and the fungal ITS genes were sequenced using Illumina MiSeq platform (Illumina, United States). The raw sequences were analyzed using the QIIME (v1.9.1) pipeline (Caporaso et al., 2010). Specifically, we trimmed each sequence using the paired-end mode of Trimmomatic (v0.39), with an average Phred quality >25 within sliding window of a four–base pair (bp), and discard the filtered reads shorter than 250 bp (Bolger et al., 2014). Then, the filtered high-quality sequences were clustered into operational taxonomic units (OTUs) at 97% similarity using the UCLUST algorithm (Edgar, 2010).

### Statistical analysis

First, we performed non-metric multidimensional scaling (NMDS) based on Bray–Curtis dissimilarity of bacterial and fungal communities to visualize their variations across different planting patterns. We further used pairwise *t*-tests to examine the significance of differences for various characteristics such as soil properties, the relative abundance of bacterial and fungal phyla, alpha diversity, crop nutrients, microbial biomass, and enzymes activities. We quantified alpha diversity using species richness, Shannon, and evenness for the bacterial and fungal communities. The relationships between ecosystem functions and the module diversity of bacteria and fungi were explored with linear models. In addition, we determined the relationships between microbial diversity and ecosystem functions by Pearson correlation analysis. The analyses of non-metric multidimensional scaling (NMDS) were performed using the R package vegan V2.5-6 (Fan et al., 2020a).

Second, we considered ecosystem functions in terms of both individual function and ecosystem multifunctionality (EMF). We categorized the 18 ecosystem functions into four functional groups: soil nutrients, crop nutrients, microbial biomass, and enzyme activities. For soil nutrients, we considered the concentrations of TOC, TN, TP, K, AN, AP, and AK. For crop nutrients, we considered the concentrations of grain yield, straw yield, grain N, grain P, straw N, and straw P. For microbial biomass, we examined the concentrations of the microbial biomass carbon and nitrogen. Finally, for enzyme activities, we considered the concentrations of catalase, phosphatase, and urease. For EMF, we used an averaging approach to estimate the average value of multiple functions observed in a given sample so as to collapse multifunctionality into a single metric. The analyses of ecosystem multifunctionality (EMF) were performed using the R package multifunc V0.9.4 (Hu et al., 2020).

Third, the co-occurrence network was constructed based on the Spearman correlation matrix (Langfelder and Horvath, 2012). We only focused on the modules with bacterial and fungal OTUs number more than 58 and 10, respectively (Supplementary Figure 1). Then all pairwise Spearman correlations between ecosystem functions and the richness or relative abundance of all modules were examined. The richness and relative abundance of each module was calculated by averaging the standardized richness and relative abundance (*z*-score) of the species within each ecological cluster. Ecological clusters (modules) represent important ecological units that play an important role in identifying highly connected taxa (Fan et al., 2020a,b). The analyses of co-occurrence network were performed using the R package WGCNA V1.71 (Fan et al., 2020a).

Finally, we quantified the magnitude and direction (i.e., positive or negative) of intercropping effects on soil physicochemical properties, microbial alpha diversity, and ecosystem functions. We used the Cohen’s *d* as the effect-size metric.


$$d=\frac{\bar{M_{t}}-\bar{M_{c}}}{\sqrt{\frac{S_{t}^{3}+S_{c}^{3}}{2}}}$$

where d is the Cohen’s *d*, $\bar{M_{t}}$ and $\bar{M_{c}}$ are the means of treatment and control groups, respectively, and $S_{t}$ and $S_{c}$ are the standard deviations of treatment and control groups, respectively. The analyses of effect-size were performed using the R package metafor V3.8.1 (Hu et al., 2021).

## Results

### Effects of intercropping on soil physicochemical properties and ecosystem functions

**Figure 2:**
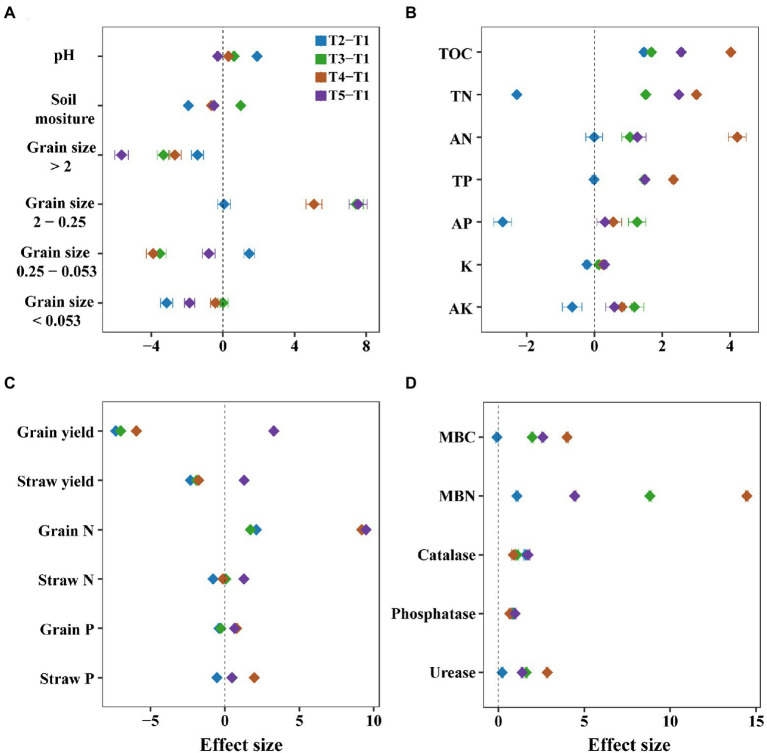
Comparison of the effects of monocropping and intercropping on soil physicochemical properties and ecosystem functions. We considered soil physicochemical properties **(A)**, soil nutrients **(B)**, crop nutrients **(C)** and the microbial biomass carbon (MBC) and nitrogen (MBN) and enzymes activities of urease, phosphatase, and catalase **(D)**. These soil physicochemical properties included soil moisture, pH, the grain sizes of aggregate <0.053, 0.25–0.053, 2–0.025, and >2 mm; soil nutrients included total organic carbon (TOC), total nitrogen (TN), total phosphorus (TP), total potassium (TK), available nitrogen (AN), available phosphorus (AP), available potassium (AK); crop nutrients included straw yield, grain yield, straw nitrogen (Straw N), straw phosphorus (Straw P), grain nitrogen (Grain N) and grain phosphorus (Grain P). The Cohen’s *d* is used as the effect size in our meta-analysis, and error bars denote the 95% confidence interval. T1, maize monocropping; T2, maize/sesame intercropping; T3, maize/peanut intercropping; T2, maize/soybean intercropping; T2, maize/sweet potato intercropping.

Generally, intercropping changed the soil physicochemical properties such as soil nutrients and aggregates. For instance, the measured soil nutrients generally increased in most intercropping systems (Figure 2B). Among them, the maize/soybean intercropping significantly elevated the content of total organic carbon, total nitrogen, available nitrogen, and total phosphorus (*p* < 0.05, Supplementary Figure 2). In addition, the percentage of grain size 2–0.25 mm significantly increased (*p* < 0.05) compared to the percentage of grain size <0.053 mm, 0.053–0.25 mm, and >2 mm in most intercropping systems (Figure 2A), especially in maize/sweet potato intercropping (*p* < 0.001, Supplementary Figure 3), with the effect size Cohen *d* of 7.54 (Supplementary Table 1). In addition, there was no significant effect on pH and soil moisture in most intercropping treatments compared to the monocropping (Figure 2A and Supplementary Figure 3).

Intercropping also improved the ecosystem functions such as grain nitrogen, enzyme activities, and microbial biomass carbon and nitrogen. Although grain and straw yield significantly decreased after intercropping, except for the maize/sweet potato intercropping, we found that intercropping had a positive effect on grain nitrogen, especially in maize/soybean and maize/sweet potato intercropping (*p* < 0.05), with the Cohen *d* of 9.19 and 9.47, respectively (Figure 2C and Supplementary Figure 4).

Soil enzymes, including catalase, phosphatase, and urease, increased among the four intercropping systems examined. The activities of urease were significantly higher in the maize/soybean intercropping system than in the monocropping systems (*p* < 0.05), with the effect size Cohen *d* of 2.84 (Figure 2D and Supplementary Figure 5). In addition, compared to the maize monocropping system, soil microbial biomass carbon and nitrogen significantly increased in most intercropping systems (*p* < 0.05, Supplementary Figure 4), especially in maize/soybean intercropping, with the Cohen *d* of 3.99 and 14.45, respectively (*p* < 0.01, Supplementary Table 1 and Supplementary Figure 5). Notably, microbial biomass nitrogen showed greater variation across these intercropping systems than microbial biomass carbon (*p* < 0.001, Figure 2D and Supplementary Figure 5).

### Effects of intercropping on microbial diversity

**Figure 3:**
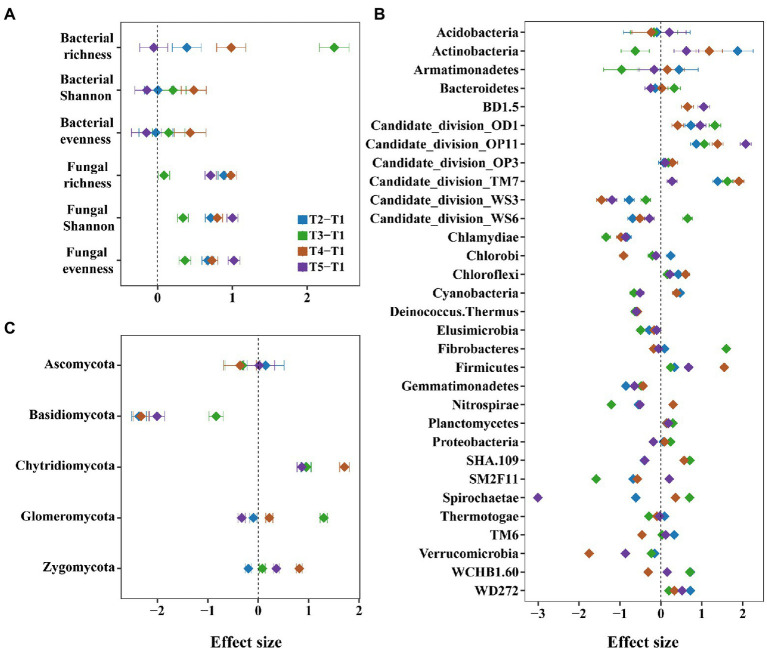
The effects of monocropping and intercropping on soil microbial diversity and communities. We considered the microbial diversity such as species richness, Shannon and evenness of bacteria and fungi **(A)**, and the microbial community such as relative abundance of bacterial **(B)** and fungal phyla **(C)**.

The alpha diversity of bacteria and fungi was higher in intercropping than in monocropping systems. For example, the species richness, Shannon index, and evenness of bacteria increased in most treatments after intercropping (Figure 3A). Among them, species richness of bacteria increased in all treatments, especially in maize/peanut intercropping, with the Cohen *d* of 2.36 (*p* < 0.05, Supplementary Table 1 and Supplementary Figure 6). Consistently, the species richness, Shannon index, and evenness of fungi increased in all intercropping systems (*p* < 0.05, Figure 3A).

For bacterial community, the relative abundance of Actinobacteria increased in most intercropping systems, especially in maize/sesame intercropping systems (Figure 3B and Supplementary Figures 7, 8). For fungal community, the intercropping systems had an increasing trend for the relative abundance of Chytridiomycota, but a decreasing trend for Basidiomycota (Figure 3C). Among them, the relative abundance of Basidiomycota was significantly decreased in the maize/sesame and maize/soybean intercropping systems (*p* < 0.05, Supplementary Figure 9).

### Effects of intercropping on the relationships between microbial diversity and ecosystem functions

**Figure 4:**
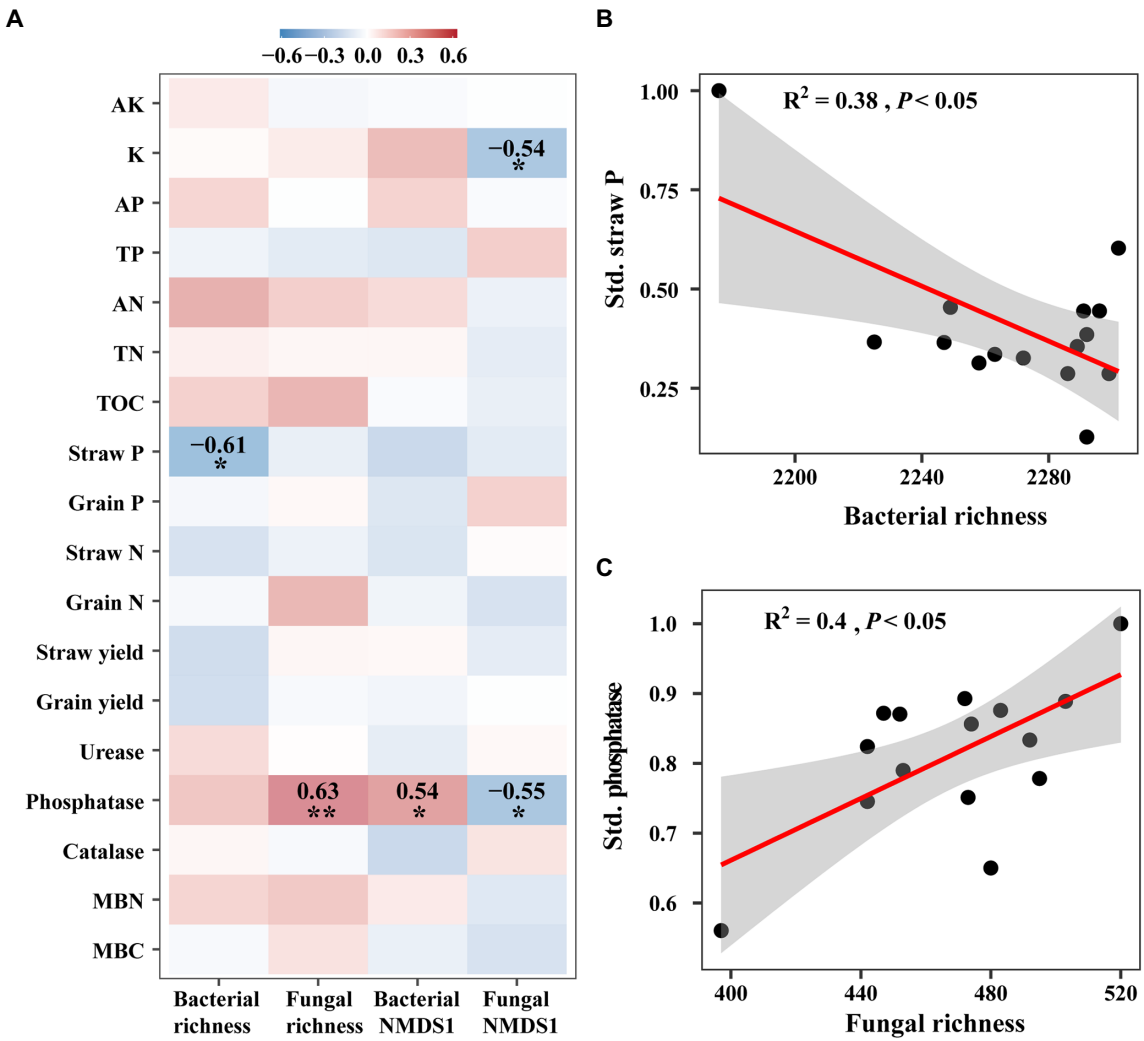
The relationships between ecosystem functions and microbial diversity. **(A)** Heatmap of the Spearman correlations between bacterial and fungal richness and composition with ecosystem functions. These functions include available potassium (AK), potassium (K), available phosphorus (AP), total phosphorus (TP), available nitrogen (AN), total nitrogen (TN), total organic carbon (TOC), straw yield, grain yield, straw phosphorus (P), grain phosphorus, straw nitrogen (N) and grain nitrogen, urease, phosphatase, and catalase, microbial biomass carbon (MBC) and nitrogen (MBN). **(B)** Regression relationship between straw phosphorus (Straw P) and bacterial richness. **(C)** Regression relationship between phosphatase and fungal richness. Red and blue colors represent positive and negative correlation, respectively. ^*^p < 0.05; ^**^p < 0.01.

We examined the relationships between microbial diversity and ecosystem functions with correlation analyses and linear models, and found that they showed significant relationships mainly for ecological clusters (that is, network modules). For overall diversity, the species richness of bacteria showed a negative correlation with straw phosphorus, while fungal richness was positively related to phosphatase (*p* < 0.05, Figures 4B,C). Bacterial composition represented by the first axis of NMDS had a positive correlation on phosphatase, whilst the first axis of fungal NMDS showed a negative correlation on potassium and phosphatase (*p* < 0.05, Figure 4A). However, for module diversity, the species richness and relative abundance of most bacterial and fungal modules showed significant correlations with individual functions. For example, the species richness of bacterial modules 4 and 7, showed significantly negative correlations with straw phosphorus (*p* < 0.001, Supplementary Figure 10A). The relative abundance of bacterial module 2 significantly negatively correlated with several functions such as grain nitrogen (*p* < 0.001, Supplementary Figure 10B).

**Figure 5:**
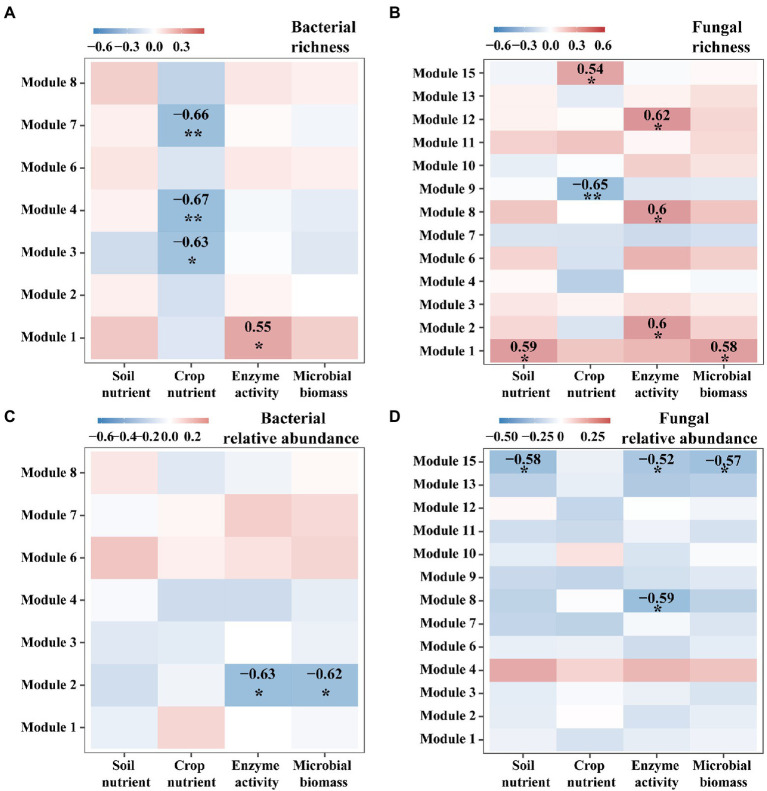
The heatmap for Spearman relationships between ecosystem multifunctionality and the characteristics of microbial modules. We considered four groups of ecosystem multifunctionality such as soil nutrient, crop nutrient, enzyme activity and microbial biomass. We considered two characteristics for each module of bacteria **(A,C)** or fungi **(B,D)**: Species richness **(A,B)** and the relative abundance **(C,D)**.

We further examined network modules of bacteria and fungi for their relationships with ecosystem multifunctionality. For instance, species richness of bacterial modules 3, 4, and 7 showed significantly negative correlations with crop nutrients (*p* < 0.05, Figure 5A), but species richness of fungal modules 2, 7, and 11 showed positive correlations with enzyme activities (*p* < 0.05), and that of modules 8 and 13 showed negative and positive correlations with crop nutrients, respectively (*p* < 0.05, Figure 5B). For relative abundance, bacterial module 2 and fungal module 13 showed significant negative correlations with most functions (*p* < 0.05, Figures 5C,D).

## Discussion

Using “metafor” package to calculated effect size for the field experiments of cropping systems in maize fields, we found that the intercropping generally enhanced microbial diversity and ecosystem functions, and affected the linkages between these two components. Moreover, intercropping altered microbial composition and resulted in higher microbial richness and ecosystem functions such as soil nutrients, crop nutrients, enzyme activity, MBC, and MBN. These findings highlighted the importance of intercropping in maintaining microbial community diversity and ecosystem functions, and provided evidence to improve soil fertility by regulating cropping systems in the agroecosystem. These knowledges are important to highlight that intercropping could improve soil fertility to alleviate the on-going issue of soil deterioration.

Intercropping significantly increased grain nitrogen, but not the yields of grain and straw. This result is in line with that of Fossati et al. (1993), showing that grain yield is negatively correlated with grain nitrogen concentration, which could be explained by the fact that nitrogen use efficiency is associated with yield variation (Triboi et al., 2006). Further explanation could be that the spacing of intercropping of crops affects the interspecific interaction and yield performance (Li et al., 1999; Ren et al., 2016; Raza et al., 2020). For instance, when the rotation strip width and sowing width were increased to 14 and 6 cm in winter wheat/white clover intercropping, respectively, the interspecific interactions could be enhanced and lead to elevated grain yield and nitrogen uptake (Thorsted et al., 2006).

We also found that intercropping systems improved the physicochemical properties of the soils, especially in soil aggregates. Specifically, the grain size 2–0.25 mm of soil aggregates (that is, macroaggregates) was positively associated with intercropping, whereas the grain size 0.25–0.053 mm of soil aggregates (that is, microaggregates) was negatively associated with intercropping. This finding agrees with previous literature which shows that intercropping significantly increases macroaggregates (that is, >0.25 mm) compared to monocropping (Tian et al., 2019). Such changes in soil aggregates can affect soil health by improving organic matter content and stability. For instance, organic matter of macroaggregates is more stable and shows higher concentrations than microaggregates (Cambardella and Elliott, 1993). The changing proportion of macroaggregates and microaggregates in agricultural fields could alter soil biological activity and nutrient retention (Xiao et al., 2021).

We further found that microbial biomass carbon and nitrogen, urease activity, and most nutrients were significantly elevated after intercropping. These results are in agreement with a previous study of Zhou et al. (2011), and could be explained by three non-exclusive explanations. First, the complex crop composition of intercropping increases residues, thereby enhancing soil microbial biomass (Bichel et al., 2017; Sekaran et al., 2020). Second, intercropping affects nutrient mobilization such as increasing soil organic carbon in the inter-rooted soil, which could improve microbial biomass carbon and nitrogen (Inal et al., 2007; Singh et al., 2021). Third, intercropping can improve urease activity *via* increasing soil microbial population and affecting soil phenolic allelopathic substances of crop root exudates (Dai et al., 2012; Li et al., 2012; Xiao et al., 2012). Notably, the most significant increase, such as in microbial biomass carbon and nitrogen, happened for maize/soybean intercropping. This is understandable as the soybean is a legume crop with nitrogen-fixing nodules caused by the interaction of roots with a beneficial soil microorganism, Rhizobium, and could thereby provide the supply of nitrogen regardless of fertilization (Rivest et al., 2010; Dyer et al., 2012; Wang et al., 2016).

Furthermore, there could also be a significant difference in microbial diversity after intercropping (Pang et al., 2021). For example, mulberry/alfalfa intercropping increases the richness and diversity of bacterial community by affecting the content of soil total carbon, available potassium, and phosphate (Zhang et al., 2018). Morus alba/Lespedeza bicolor intercropping significantly elevates the evenness and diversity of fungal community by affecting the contents of soil total carbon, nitrogen, and phosphate (Liu J. et al., 2022). Correspondingly, our results also observed that maize/peanut intercropping system can significantly increase bacterial richness. This phenomenon may result from the following two reasons. First, peanut has a nitrogen fixation effect, and thereby can influence the distribution of nitrogen in soils and further microbial composition (Guo et al., 2020; Han et al., 2022). Second, maize/peanut intercropping can promote the population of microorganisms associated with nitrogen-fixing by affecting the structure and functions of microorganisms in rhizosphere soils (Chen et al., 2018).

Finally, compared to overall microbial diversity, we found a stronger relationship between ecosystem functions and the species richness or relative abundance of microbial ecological modules. For instance, each module showed varying effects on ecosystem functions, which collectively indicates the importance of microbial diversity of ecological modules in maintaining ecosystem functioning after intercropping. This is partly consistent with previous studies in other agriculture ecosystems. For instance, ecological modules based on soil microbial phylotypes in response to precipitation influence soil carbon or nitrogen mineralization rates under either the dry or wet conditions in a typical semi-arid steppe, thereby affecting ecosystem functions (Wu et al., 2020). The application of organic fertilizers results in various ecological clusters of microbial species, which are relevant to essential ecological functions such as nutrient cycling and organic degrading (Wang et al., 2022). Such results could be explained by the fact that compare to the whole community, soil functions may be conducted by specialized microbes which were expected to build ecological clusters to maintain vital ecosystem functions (Harvey et al., 2017; Li et al., 2020).

## Conclusion

Our study described the differences in soil physicochemical properties, ecosystem functions, and microbial diversity between monocropping and intercropping systems, as well as their effects on the link between microbial diversity and ecosystem functions. Specifically, in terms of soil physicochemical properties, most intercropping systems significantly increases soil macroaggregates and beneficial for soil physical structure. In terms of ecosystem functions, most intercropping had significantly positive effects on urease activity, microbial biomass carbon and nitrogen. In terms of diversity, the maize/peanut intercropping significantly elevated the species richness of bacteria. We further found that rather than the whole microbial diversity, the microbial diversity of ecological clusters showed important roles in maintaining ecosystem functions. We thus expect that intercropping not only contributes to improve soil quality in agricultural ecosystems, but also provides further insights into the relationships between soil microbial diversity and ecosystem functions.

## Data availability statement

The datasets presented in this study can be found in online repositories. The names of the repository/repositories and accession number(s) can be found in the article/Supplementary material.

## Author contributions

XX: writing—original draft, formal analysis, and writing—review and editing. LH: data curation, formal analysis, and visualization. HC: sampling and laboratory analyses. JW: writing—review and editing. AH and YZ: conceptualization, formal analysis, and writing—review and editing. All authors contributed to the article and approved the submitted version.

## Funding

This study was supported by National Natural Science Foundation of China (42077052) and Scientific Research Fund of Hunan Provincial Education Department of China (21B0191).

## Conflict of interest

The authors declare that the research was conducted in the absence of any commercial or financial relationships that could be construed as a potential conflict of interest.

## Publisher’s note

All claims expressed in this article are solely those of the authors and do not necessarily represent those of their affiliated organizations, or those of the publisher, the editors and the reviewers. Any product that may be evaluated in this article, or claim that may be made by its manufacturer, is not guaranteed or endorsed by the publisher.
