# Supplementary material for: Intercropping enhances microbial community diversity and ecosystem functioning in maize fields
Source: Front Microbiol. 2023 Jan 4;13:1084452. doi: 10.3389/fmicb.2022.1084452 (PMC9846038; doi:10.3389/fmicb.2022.1084452)
Supplement: Supplementary file 1 [file Data_Sheet_1.docx]

**Supplementary Figures**


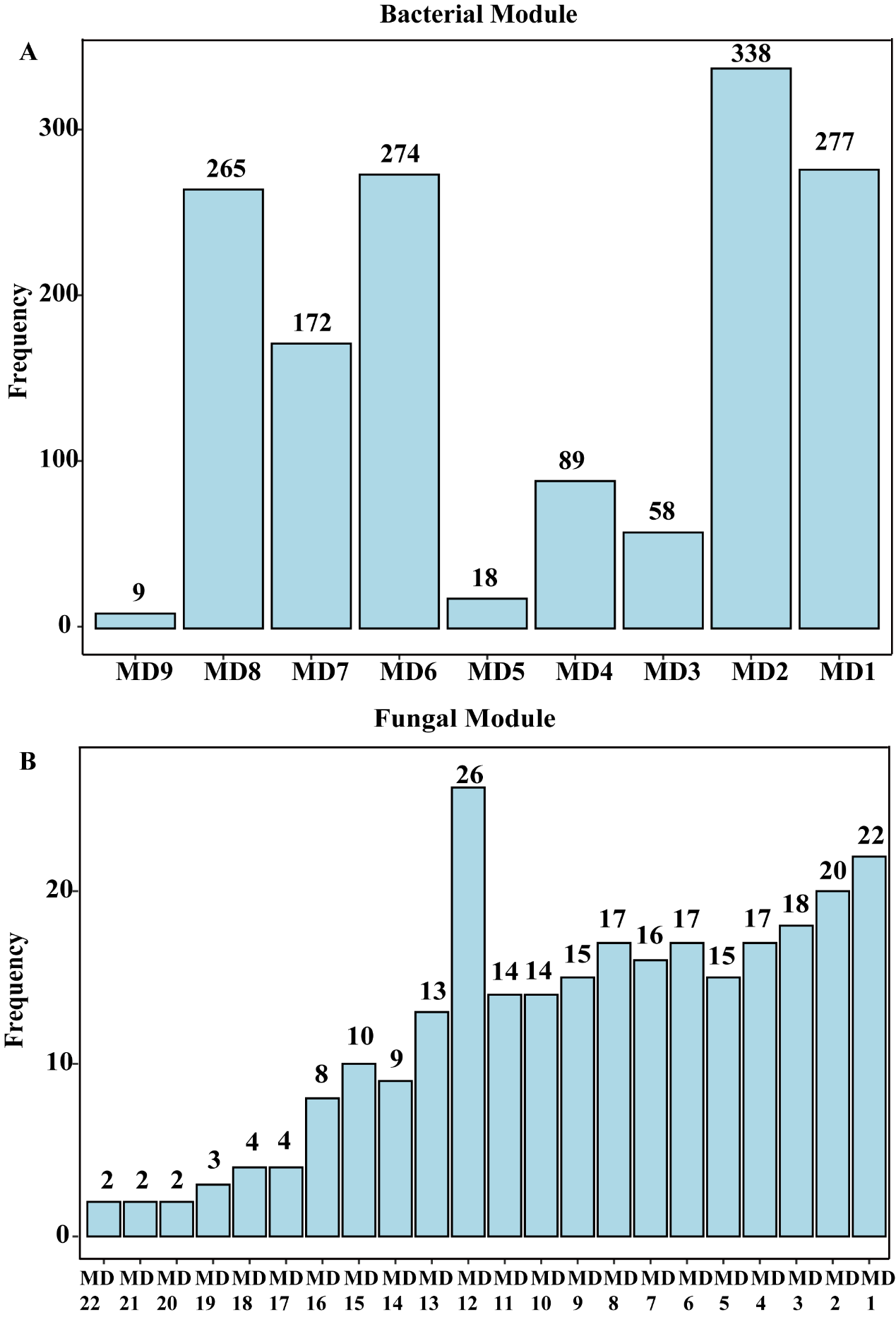
 **Supplementary Figure 1.** Bacterial **(A)** and fungal **(B)** community are divided into the nine (Modules 1–9) and twenty-two (Modules 1–22) ecological clusters, respectively, and each module (MD) contains different numbers of operational taxonomic units (OTUs).


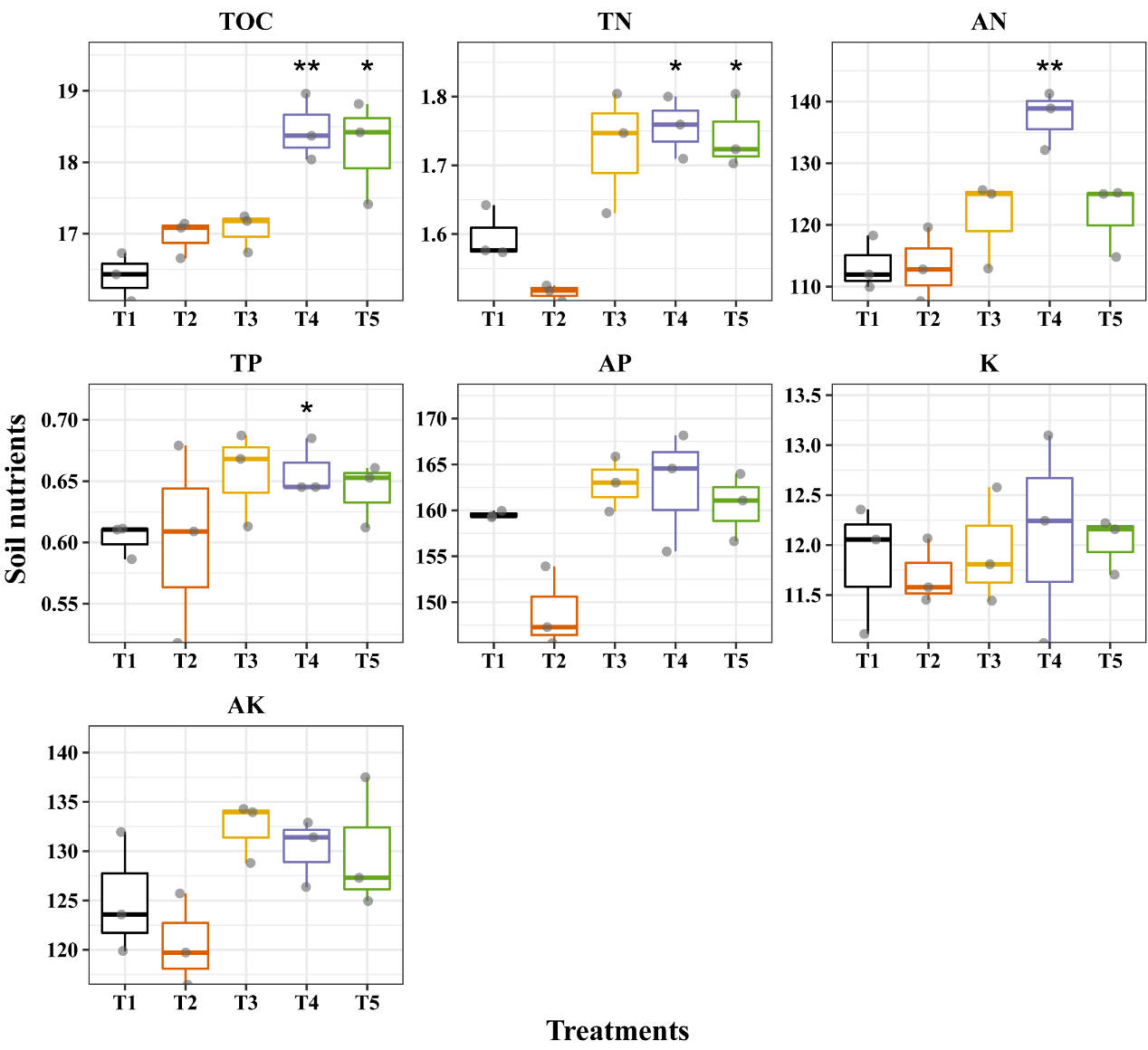


**Supplementary Figure 2.** The effects of intercropping on soil nutrient of total organic carbon (TOC), total nitrogen (TN), total phosphorus (TP), total potassium (TK), available nitrogen (AN), available phosphorus (AP), available potassium (AK). Boundaries of boxes indicate the first and third quartiles, and lines and squares within boxes represent the median and average, respectively. Whiskers indicate the 10th and 90th percentiles, and outliers are shown as dots. T1, maize monocropping; T2, maize/sesame intercropping; T3, maize/peanut intercropping; T2, maize/soybean intercropping; T2, maize/sweet potato intercropping. **P* < 0.05; ***P* < 0.01; ****P* < 0.001.


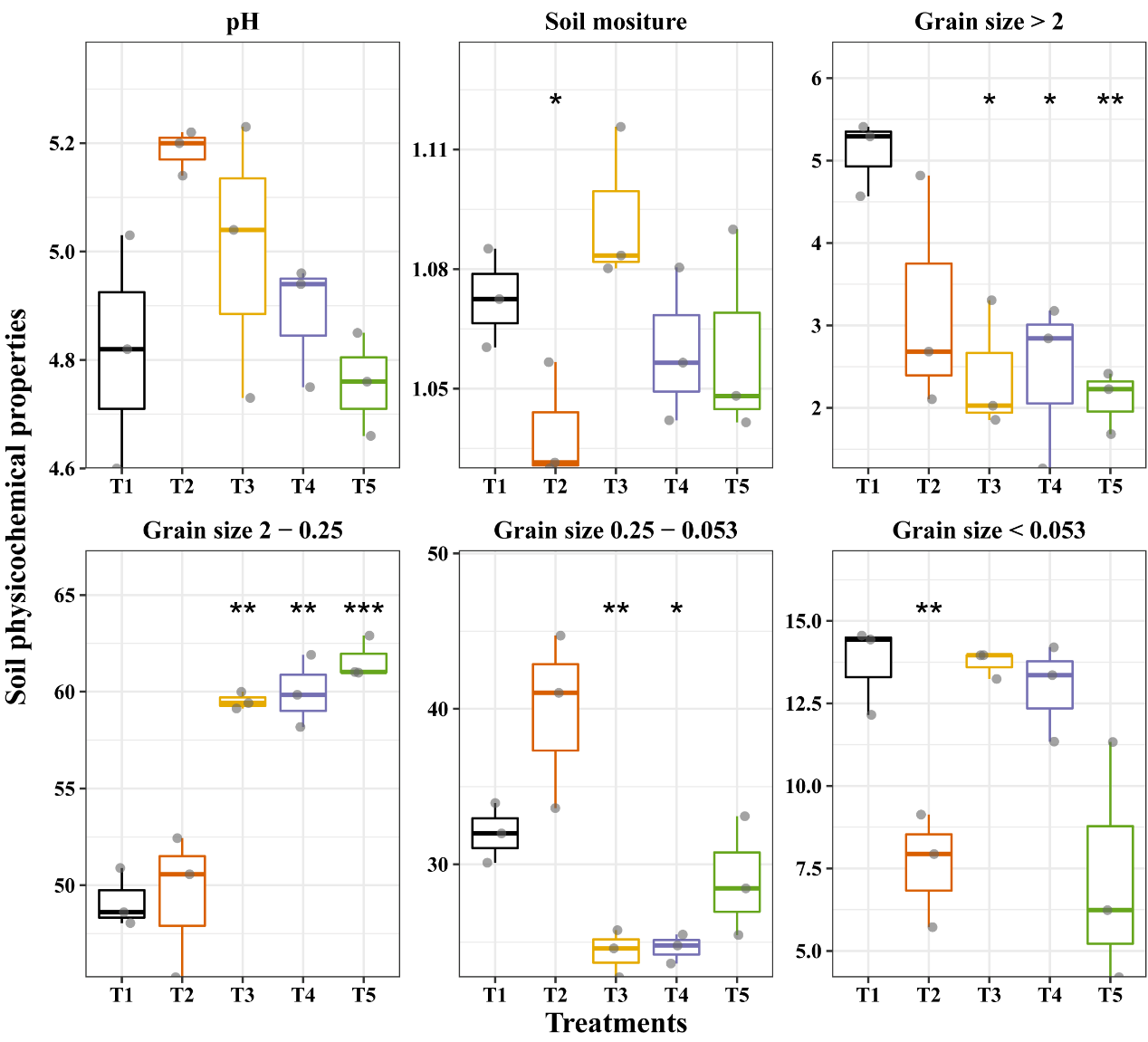


**Supplementary Figure 3.** The effects of intercropping on soil properties of pH, soil moisture, the grain sizes of aggregate < 0.053 mm, 0.25 - 0.053 mm, 2 - 0.025 mm, and > 2 mm.
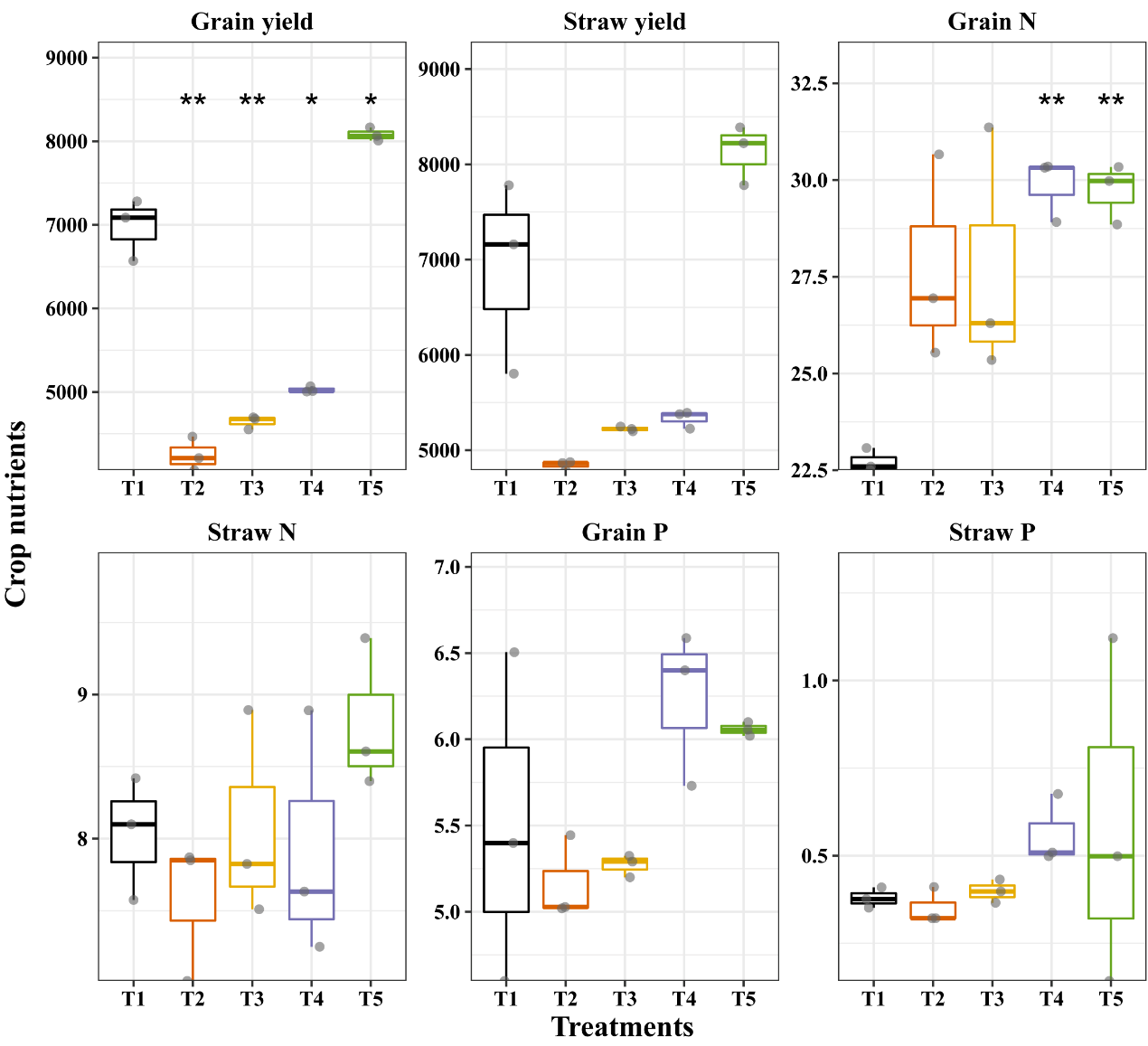


**Supplementary Figure 4.** The effects of intercropping on crop nutrients of straw yield, grain yield, straw nitrogen (Straw N), straw phosphorus (Straw P), grain nitrogen (Grain N) and grain phosphorus (Grain P).


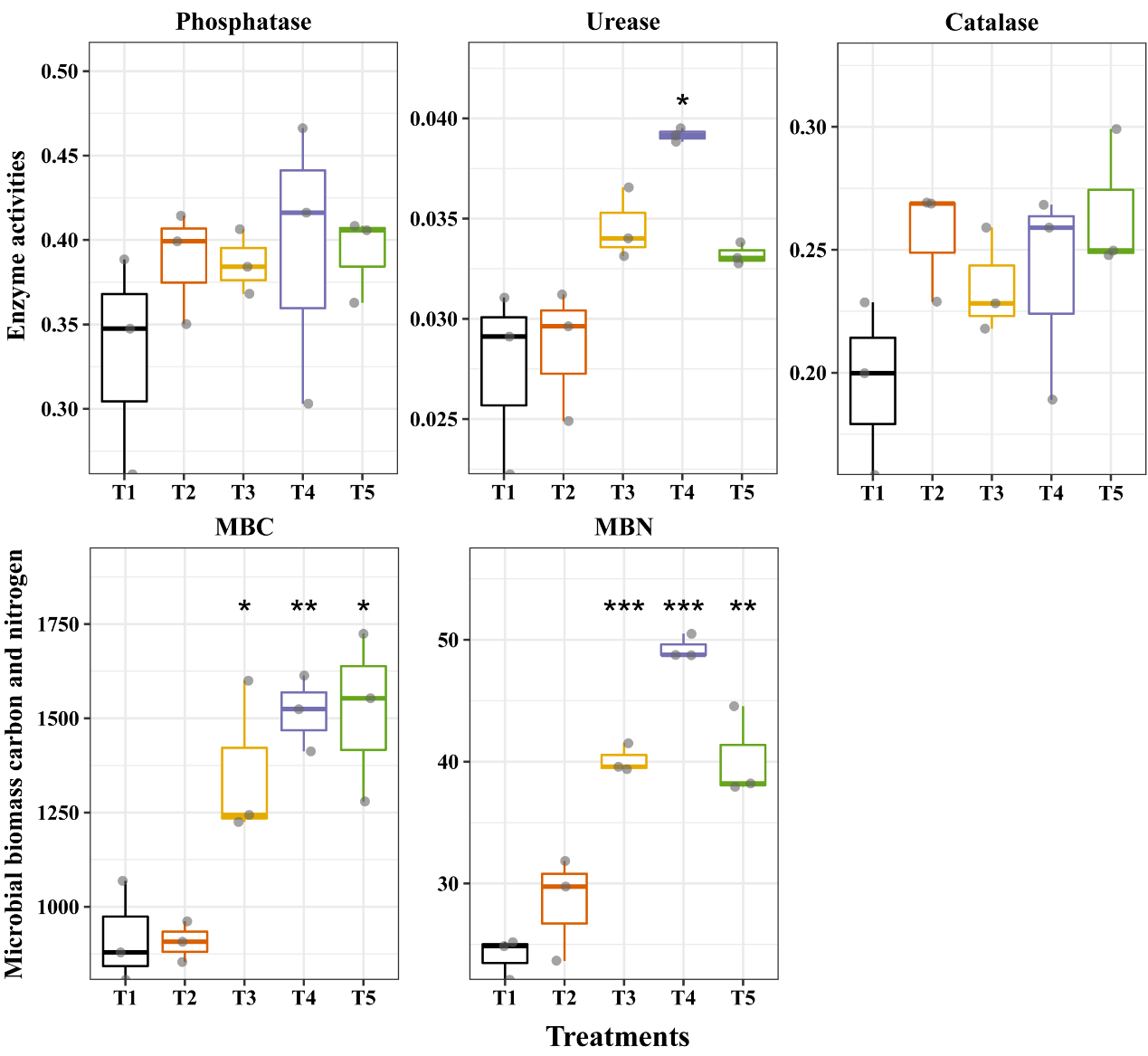


**Supplementary Figure 5.** The effects of intercropping on microbial biomass carbon (MBC) and nitrogen (MBN) and enzymes activities of urease, phosphatase, and catalase.


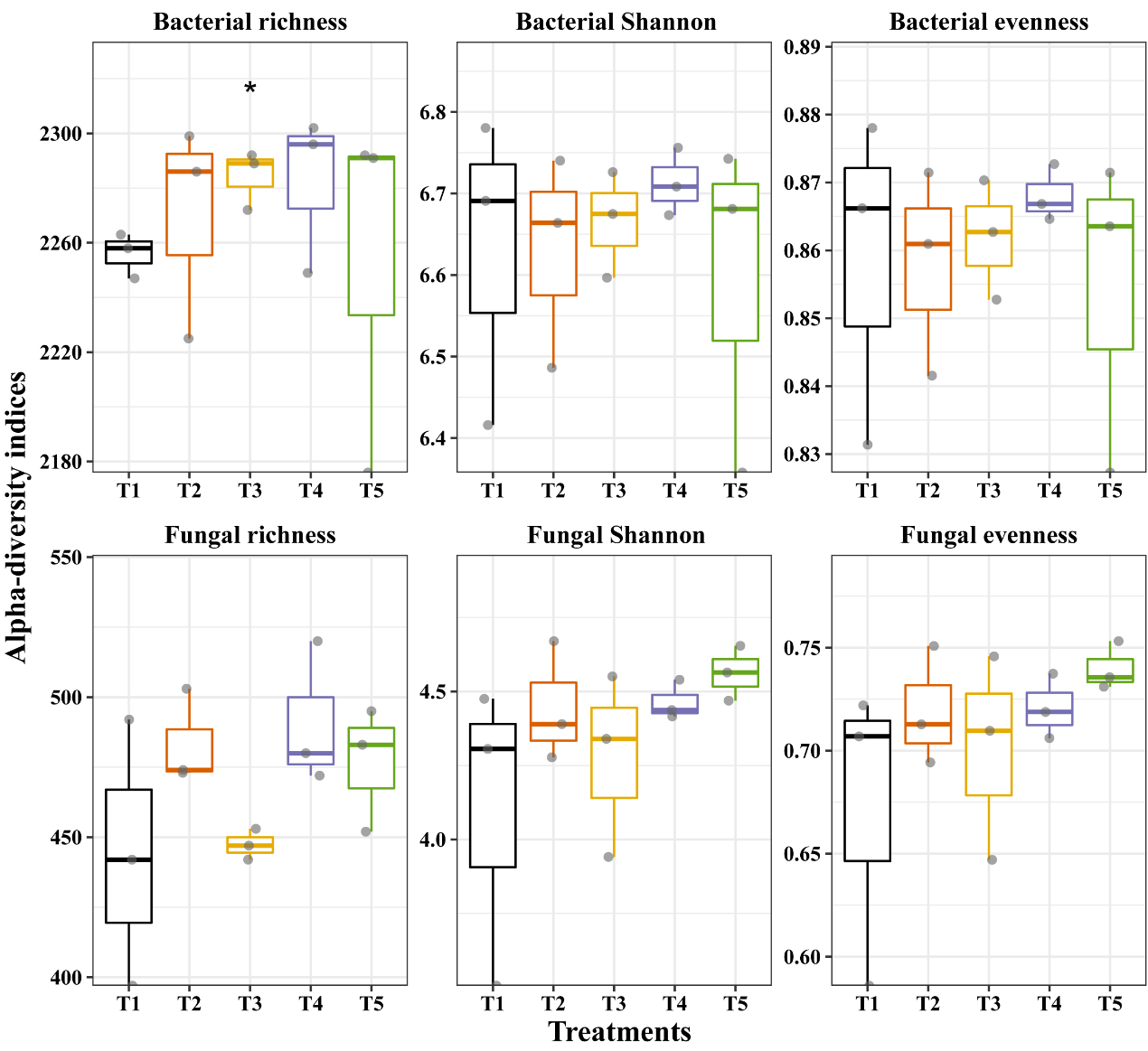


**Supplementary Figure 6.** The effects of intercropping on soil microbial diversity. We considered the microbial alpha diversity indices of richness, Shannon, evenness.


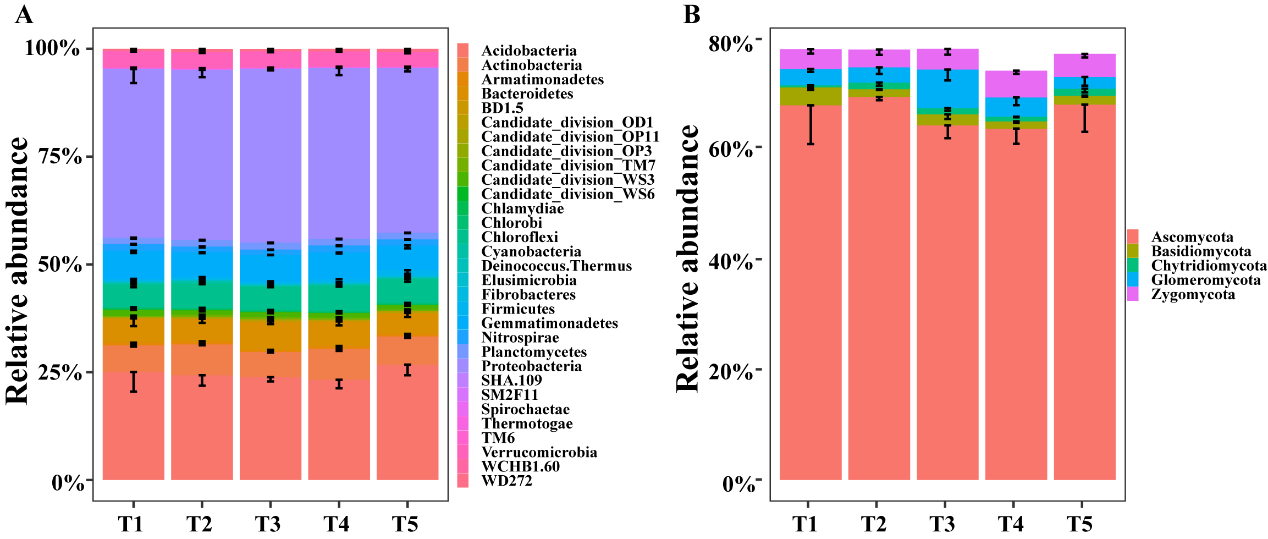


**Supplementary Figure 7.** Effects of intercropping on relative abundance of soil microbial communities. **(A)** Bacterial community; **(B)** fungal community. T1, maize monocropping; T2, maize/sesame intercropping; T3, maize/peanut intercropping; T2, maize/soybean intercropping; T2, maize/sweet potato intercropping.


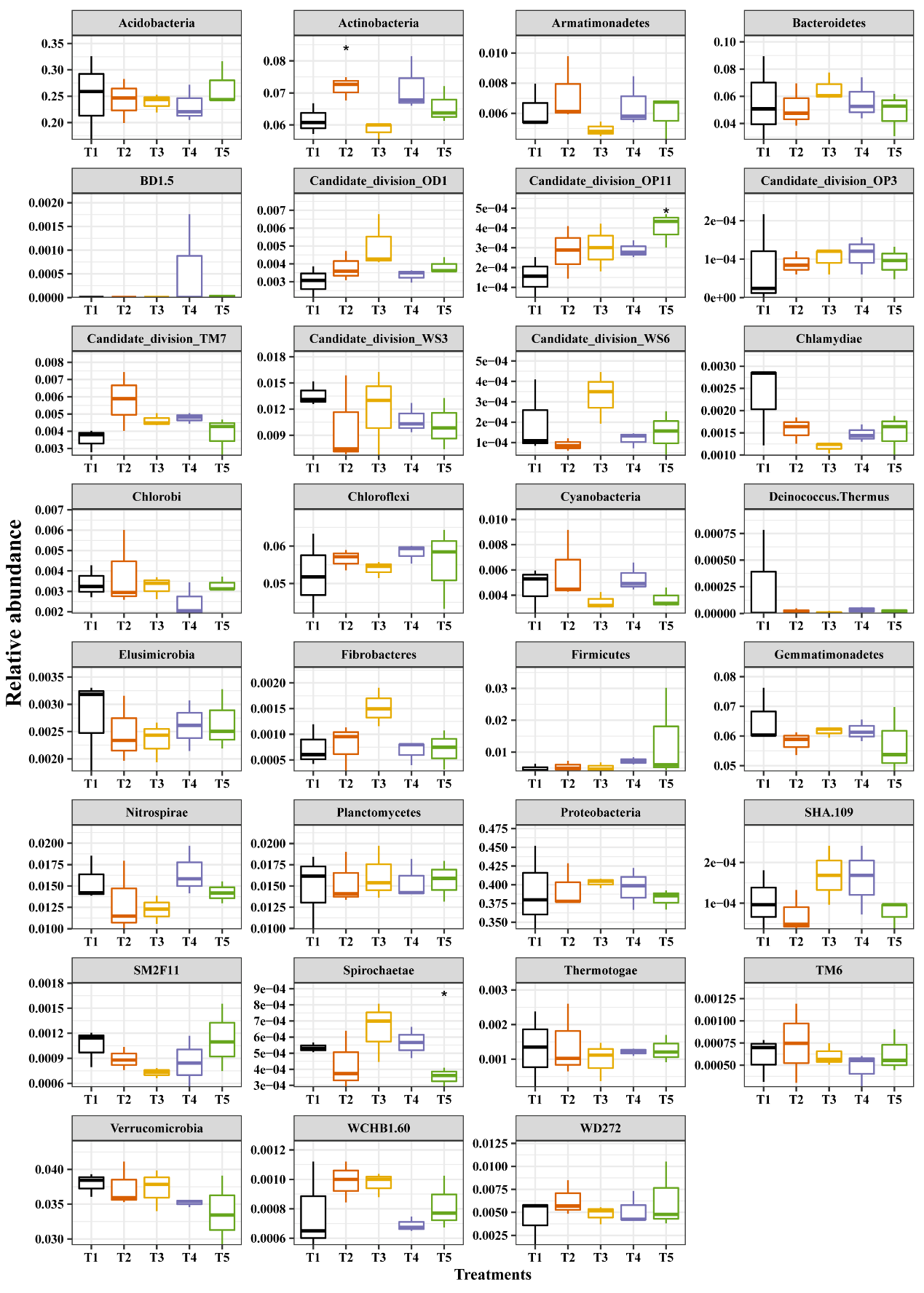
 **Supplementary Figure 8.** The effects of intercropping on relative abundance of bacterial phyla.


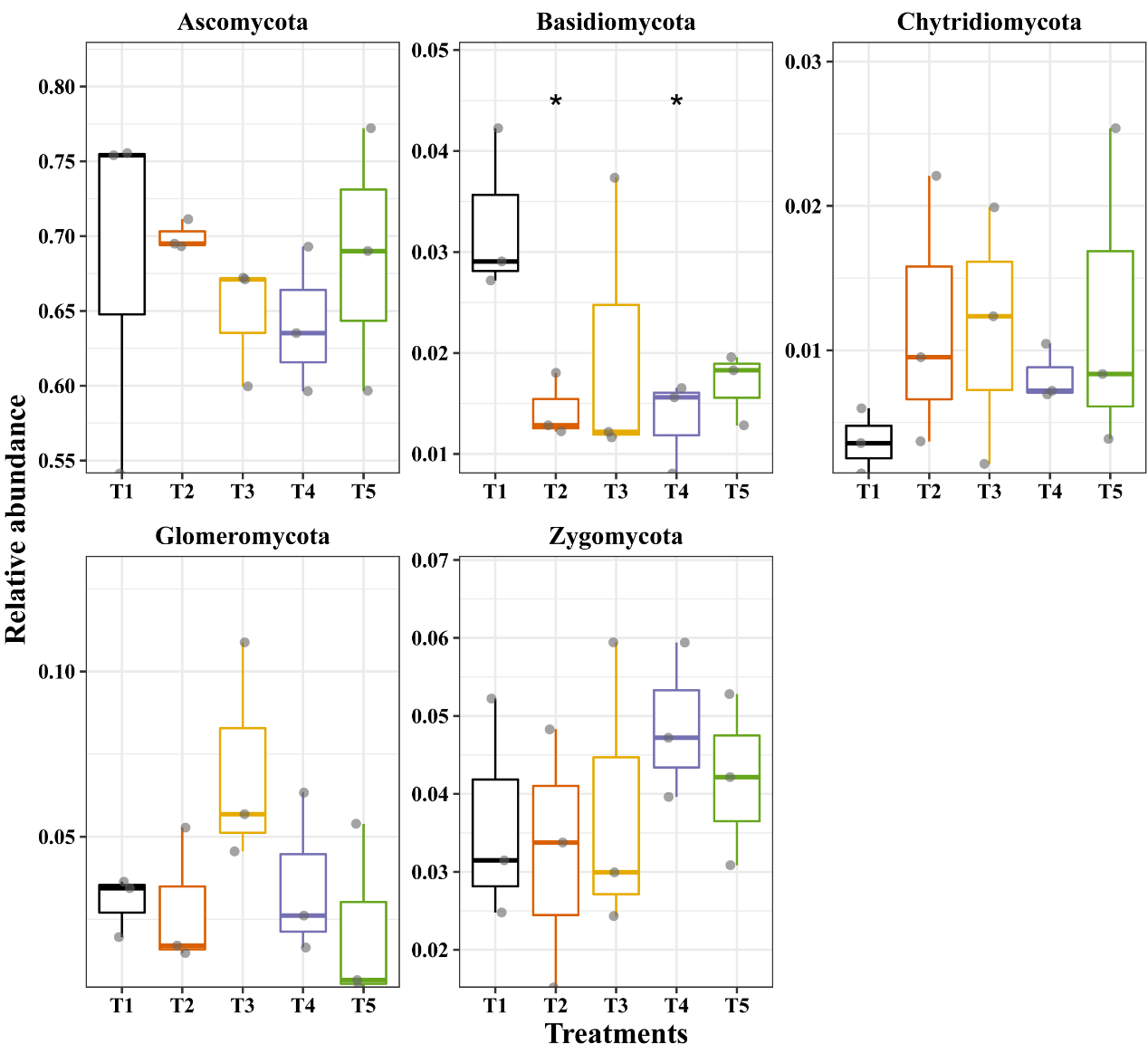
 **Supplementary Figure 9.** The effects of intercropping on relative abundance of main fungal phyla.


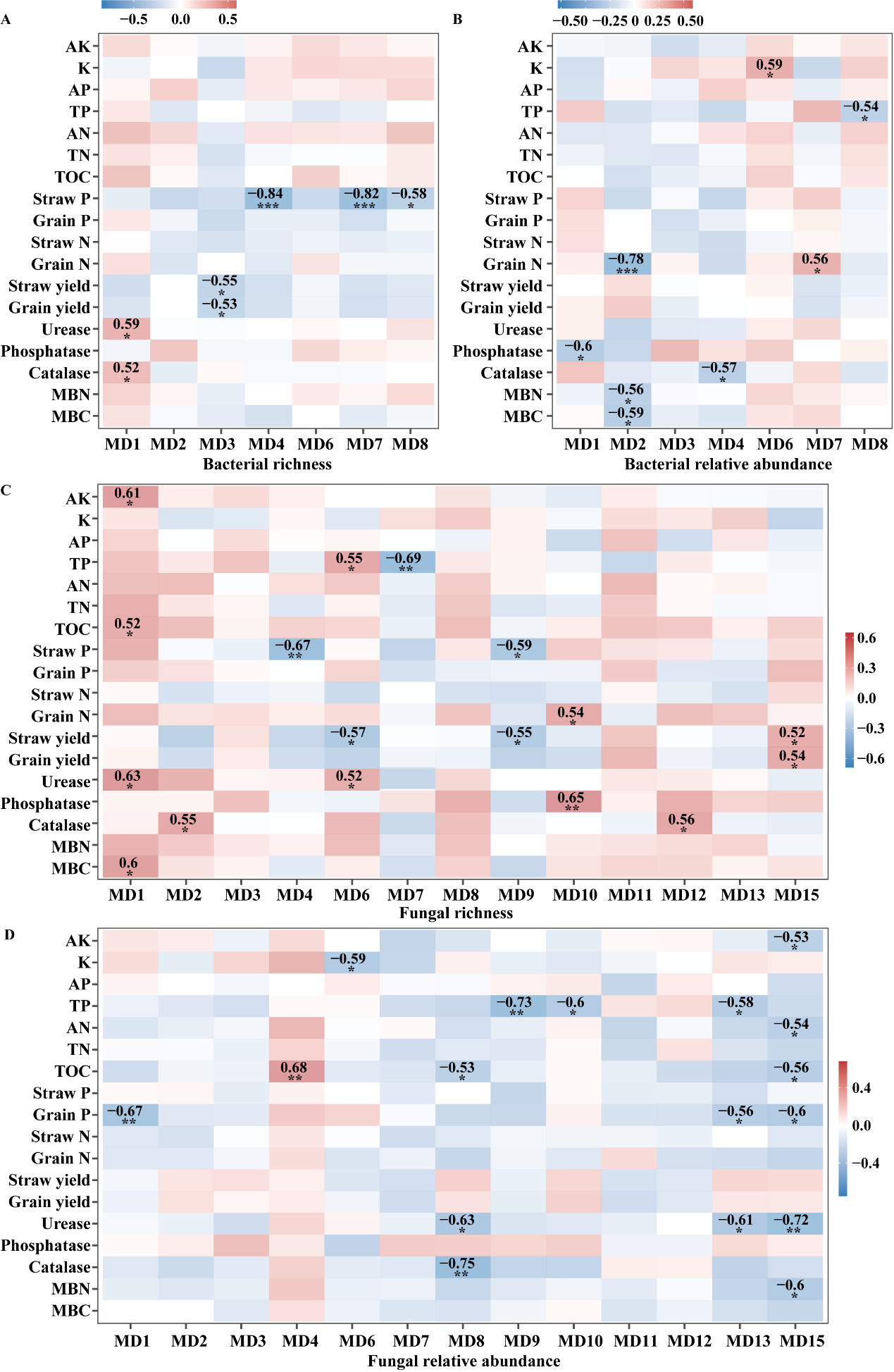
 **Supplementary Figure 10.** Spearman correlations between ecosystem functions and the richness **(A)** and relative abundance **(B)** of bacterial modules (MD), and richness **(C)** and relative abundance **(D)** of fungal modules (MD). We included ecosystem functions of available potassium (AK), potassium (K), available phosphorus (AP), total phosphorus (TP), available nitrogen (AN), total nitrogen (TN), total organic carbon (TOC), straw yield, grain yield, straw phosphorus (Straw P), grain phosphorus (Grain P), straw nitrogen (Straw N) and grain nitrogen (Grain N), urease, phosphatase, and catalase, microbial biomass carbon (MBC) and nitrogen (MBN). **P* < 0.05; ***P* < 0.01; ****P* < 0.001.

| Group | Variable | Control | Treatment | Mean  _control_ | Mean  _treatment_ | Sd  _control_ | Sd  _treatment_ | yi | vi |
| --- | --- | --- | --- | --- | --- | --- | --- | --- | --- |
| T2-T1 | pH | 3 | 3 | 5.187 | 4.817 | 0.042 | 0.215 | 1.906 | 0.969 |
| T3-T1 | pH | 3 | 3 | 5.000 | 4.817 | 0.252 | 0.215 | 0.624 | 0.699 |
| T4-T1 | pH | 3 | 3 | 4.883 | 4.817 | 0.116 | 0.215 | 0.308 | 0.675 |
| T5-T1 | pH | 3 | 3 | 4.757 | 4.817 | 0.095 | 0.215 | -0.288 | 0.674 |
| T2-T1 | Soil mositure | 3 | 3 | 1.039 | 1.073 | 0.015 | 0.012 | -1.932 | 0.978 |
| T3-T1 | Soil mositure | 3 | 3 | 1.093 | 1.073 | 0.020 | 0.012 | 0.994 | 0.749 |
| T4-T1 | Soil mositure | 3 | 3 | 1.060 | 1.073 | 0.019 | 0.012 | -0.638 | 0.701 |
| T5-T1 | Soil mositure | 3 | 3 | 1.060 | 1.073 | 0.026 | 0.012 | -0.495 | 0.687 |
| T2-T1 | Grain size > 2 | 3 | 3 | 3.202 | 5.089 | 1.428 | 0.456 | -1.420 | 0.835 |
| T3-T1 | Grain size > 2 | 3 | 3 | 2.396 | 5.089 | 0.793 | 0.456 | -3.322 | 1.587 |
| T4-T1 | Grain size > 2 | 3 | 3 | 2.429 | 5.089 | 1.020 | 0.456 | -2.686 | 1.268 |
| T5-T1 | Grain size > 2 | 3 | 3 | 2.109 | 5.089 | 0.380 | 0.456 | -5.661 | 3.337 |
| T2-T1 | Grain size 2 - 0.25 | 3 | 3 | 49.417 | 49.182 | 3.729 | 1.505 | 0.066 | 0.667 |
| T3-T1 | Grain size 2 - 0.25 | 3 | 3 | 59.515 | 49.182 | 0.438 | 1.505 | 7.436 | 5.275 |
| T4-T1 | Grain size 2 - 0.25 | 3 | 3 | 59.977 | 49.182 | 1.869 | 1.505 | 5.075 | 2.813 |
| T5-T1 | Grain size 2 - 0.25 | 3 | 3 | 61.636 | 49.182 | 1.095 | 1.505 | 7.549 | 5.416 |
| T2-T1 | Grain size 0.25 - 0.053 | 3 | 3 | 39.787 | 32.013 | 5.650 | 1.920 | 1.470 | 0.847 |
| T3-T1 | Grain size 0.25 - 0.053 | 3 | 3 | 24.368 | 32.013 | 1.526 | 1.920 | -3.517 | 1.697 |
| T4-T1 | Grain size 0.25 - 0.053 | 3 | 3 | 24.628 | 32.013 | 0.939 | 1.920 | -3.899 | 1.933 |
| T5-T1 | Grain size 0.25 - 0.053 | 3 | 3 | 29.001 | 32.013 | 3.849 | 1.920 | -0.790 | 0.719 |
| T2-T1 | Grain size < 0.053 | 3 | 3 | 7.594 | 13.715 | 1.732 | 1.354 | -3.142 | 1.489 |
| T3-T1 | Grain size < 0.053 | 3 | 3 | 13.721 | 13.715 | 0.416 | 1.354 | 0.005 | 0.667 |
| T4-T1 | Grain size < 0.053 | 3 | 3 | 12.967 | 13.715 | 1.469 | 1.354 | -0.423 | 0.682 |
| T5-T1 | Grain size < 0.053 | 3 | 3 | 7.254 | 13.715 | 3.672 | 1.354 | -1.862 | 0.956 |
| T2-T1 | TOC | 3 | 3 | 16.961 | 16.405 | 0.265 | 0.337 | 1.462 | 0.845 |
| T3-T1 | TOC | 3 | 3 | 17.053 | 16.405 | 0.275 | 0.337 | 1.681 | 0.902 |
| T4-T1 | TOC | 3 | 3 | 18.457 | 16.405 | 0.467 | 0.337 | 4.023 | 2.015 |
| T5-T1 | TOC | 3 | 3 | 18.216 | 16.405 | 0.723 | 0.337 | 2.563 | 1.214 |
| T2-T1 | TN | 3 | 3 | 1.515 | 1.597 | 0.012 | 0.039 | -2.289 | 1.103 |
| T3-T1 | TN | 3 | 3 | 1.727 | 1.597 | 0.089 | 0.039 | 1.515 | 0.858 |
| T4-T1 | TN | 3 | 3 | 1.756 | 1.597 | 0.045 | 0.039 | 3.008 | 1.421 |
| T5-T1 | TN | 3 | 3 | 1.743 | 1.597 | 0.054 | 0.039 | 2.493 | 1.185 |
| T2-T1 | AN | 3 | 3 | 113.343 | 113.378 | 6.008 | 4.360 | -0.005 | 0.667 |
| T3-T1 | AN | 3 | 3 | 121.197 | 113.378 | 7.174 | 4.360 | 1.051 | 0.759 |
| T4-T1 | AN | 3 | 3 | 137.428 | 113.378 | 4.740 | 4.360 | 4.214 | 2.146 |
| T5-T1 | AN | 3 | 3 | 121.665 | 113.378 | 5.952 | 4.360 | 1.267 | 0.801 |
| T2-T1 | TP | 3 | 3 | 0.602 | 0.603 | 0.081 | 0.014 | -0.010 | 0.667 |
| T3-T1 | TP | 3 | 3 | 0.656 | 0.603 | 0.039 | 0.014 | 1.469 | 0.847 |
| T4-T1 | TP | 3 | 3 | 0.658 | 0.603 | 0.023 | 0.014 | 2.334 | 1.121 |
| T5-T1 | TP | 3 | 3 | 0.642 | 0.603 | 0.026 | 0.014 | 1.493 | 0.852 |
| T2-T1 | AP | 3 | 3 | 148.918 | 159.528 | 4.411 | 0.364 | -2.705 | 1.276 |
| T3-T1 | AP | 3 | 3 | 162.915 | 159.528 | 3.003 | 0.364 | 1.264 | 0.800 |
| T4-T1 | AP | 3 | 3 | 162.747 | 159.528 | 6.510 | 0.364 | 0.557 | 0.693 |
| T5-T1 | AP | 3 | 3 | 160.560 | 159.528 | 3.692 | 0.364 | 0.314 | 0.675 |
| T2-T1 | K | 3 | 3 | 11.699 | 11.840 | 0.325 | 0.649 | -0.220 | 0.671 |
| T3-T1 | K | 3 | 3 | 11.943 | 11.840 | 0.579 | 0.649 | 0.133 | 0.668 |
| T4-T1 | K | 3 | 3 | 12.120 | 11.840 | 1.043 | 0.649 | 0.257 | 0.672 |
| T5-T1 | K | 3 | 3 | 12.027 | 11.840 | 0.282 | 0.649 | 0.297 | 0.674 |
| T2-T1 | AK | 3 | 3 | 120.634 | 125.136 | 4.690 | 6.177 | -0.655 | 0.702 |
| T3-T1 | AK | 3 | 3 | 132.345 | 125.136 | 3.068 | 6.177 | 1.179 | 0.783 |
| T4-T1 | AK | 3 | 3 | 130.230 | 125.136 | 3.422 | 6.177 | 0.814 | 0.722 |
| T5-T1 | AK | 3 | 3 | 129.915 | 125.136 | 6.670 | 6.177 | 0.593 | 0.696 |
| T2-T1 | Grain yield | 3 | 3 | 4246.415 | 6978.892 | 203.506 | 368.551 | -7.324 | 5.136 |
| T3-T1 | Grain yield | 3 | 3 | 4642.422 | 6978.892 | 78.742 | 368.551 | -6.996 | 4.745 |
| T4-T1 | Grain yield | 3 | 3 | 5028.796 | 6978.892 | 34.956 | 368.551 | -5.944 | 3.611 |
| T5-T1 | Grain yield | 3 | 3 | 8079.496 | 6978.892 | 80.649 | 368.551 | 3.292 | 1.570 |
| T2-T1 | Straw yield | 3 | 3 | 4843.576 | 6915.436 | 46.653 | 1011.561 | -2.309 | 1.111 |
| T3-T1 | Straw yield | 3 | 3 | 5223.772 | 6915.436 | 24.701 | 1011.561 | -1.886 | 0.963 |
| T4-T1 | Straw yield | 3 | 3 | 5332.442 | 6915.436 | 92.399 | 1011.561 | -1.758 | 0.924 |
| T5-T1 | Straw yield | 3 | 3 | 8131.178 | 6915.436 | 313.261 | 1011.561 | 1.295 | 0.807 |
| T2-T1 | Grain N | 3 | 3 | 27.716 | 22.723 | 2.647 | 0.308 | 2.114 | 1.039 |
| T3-T1 | Grain N | 3 | 3 | 27.671 | 22.723 | 3.232 | 0.308 | 1.719 | 0.913 |
| T4-T1 | Grain N | 3 | 3 | 29.862 | 22.723 | 0.820 | 0.308 | 9.194 | 7.710 |
| T5-T1 | Grain N | 3 | 3 | 29.723 | 22.723 | 0.774 | 0.308 | 9.479 | 8.155 |
| T2-T1 | Straw N | 3 | 3 | 7.578 | 8.031 | 0.490 | 0.427 | -0.788 | 0.718 |
| T3-T1 | Straw N | 3 | 3 | 8.076 | 8.031 | 0.725 | 0.427 | 0.060 | 0.667 |
| T4-T1 | Straw N | 3 | 3 | 7.924 | 8.031 | 0.858 | 0.427 | -0.126 | 0.668 |
| T5-T1 | Straw N | 3 | 3 | 8.799 | 8.031 | 0.524 | 0.427 | 1.282 | 0.804 |
| T2-T1 | Grain P | 3 | 3 | 5.164 | 5.501 | 0.243 | 0.957 | -0.385 | 0.679 |
| T3-T1 | Grain P | 3 | 3 | 5.272 | 5.501 | 0.064 | 0.957 | -0.269 | 0.673 |
| T4-T1 | Grain P | 3 | 3 | 6.239 | 5.501 | 0.450 | 0.957 | 0.787 | 0.718 |
| T5-T1 | Grain P | 3 | 3 | 6.058 | 5.501 | 0.040 | 0.957 | 0.657 | 0.703 |
| T2-T1 | Straw P | 3 | 3 | 0.351 | 0.379 | 0.052 | 0.029 | -0.529 | 0.690 |
| T3-T1 | Straw P | 3 | 3 | 0.398 | 0.379 | 0.033 | 0.029 | 0.499 | 0.687 |
| T4-T1 | Straw P | 3 | 3 | 0.561 | 0.379 | 0.100 | 0.029 | 1.979 | 0.993 |
| T5-T1 | Straw P | 3 | 3 | 0.587 | 0.379 | 0.496 | 0.029 | 0.474 | 0.685 |
| T2-T1 | MBC | 3 | 3 | 907.400 | 918.047 | 53.833 | 135.564 | -0.082 | 0.667 |
| T3-T1 | MBC | 3 | 3 | 1356.185 | 918.047 | 211.114 | 135.564 | 1.971 | 0.990 |
| T4-T1 | MBC | 3 | 3 | 1516.610 | 918.047 | 100.750 | 135.564 | 3.999 | 1.999 |
| T5-T1 | MBC | 3 | 3 | 1518.958 | 918.047 | 224.326 | 135.564 | 2.587 | 1.224 |
| T2-T1 | MBN | 3 | 3 | 28.426 | 24.049 | 4.248 | 1.694 | 1.080 | 0.764 |
| T3-T1 | MBN | 3 | 3 | 40.158 | 24.049 | 1.174 | 1.694 | 8.819 | 7.147 |
| T4-T1 | MBN | 3 | 3 | 49.327 | 24.049 | 1.011 | 1.694 | 14.457 | 18.084 |
| T5-T1 | MBN | 3 | 3 | 40.234 | 24.049 | 3.745 | 1.694 | 4.443 | 2.312 |
| T2-T1 | Catalase | 3 | 3 | 0.256 | 0.196 | 0.023 | 0.035 | 1.605 | 0.881 |
| T3-T1 | Catalase | 3 | 3 | 0.235 | 0.196 | 0.021 | 0.035 | 1.079 | 0.764 |
| T4-T1 | Catalase | 3 | 3 | 0.239 | 0.196 | 0.043 | 0.035 | 0.872 | 0.730 |
| T5-T1 | Catalase | 3 | 3 | 0.266 | 0.196 | 0.029 | 0.035 | 1.725 | 0.915 |
| T2-T1 | Phosphatase | 3 | 3 | 0.388 | 0.332 | 0.034 | 0.065 | 0.857 | 0.728 |
| T3-T1 | Phosphatase | 3 | 3 | 0.386 | 0.332 | 0.019 | 0.065 | 0.897 | 0.734 |
| T4-T1 | Phosphatase | 3 | 3 | 0.395 | 0.332 | 0.084 | 0.065 | 0.669 | 0.704 |
| T5-T1 | Phosphatase | 3 | 3 | 0.392 | 0.332 | 0.026 | 0.065 | 0.968 | 0.745 |
| T2-T1 | Urease | 3 | 3 | 0.029 | 0.027 | 0.003 | 0.005 | 0.220 | 0.671 |
| T3-T1 | Urease | 3 | 3 | 0.035 | 0.027 | 0.002 | 0.005 | 1.616 | 0.884 |
| T4-T1 | Urease | 3 | 3 | 0.039 | 0.027 | 0.000 | 0.005 | 2.846 | 1.342 |
| T5-T1 | Urease | 3 | 3 | 0.033 | 0.027 | 0.001 | 0.005 | 1.389 | 0.827 |
| T2-T1 | Bacterial richness | 3 | 3 | 2270.000 | 2256.000 | 39.509 | 8.185 | 0.392 | 0.679 |
| T3-T1 | Bacterial richness | 3 | 3 | 2284.333 | 2256.000 | 10.786 | 8.185 | 2.361 | 1.131 |
| T4-T1 | Bacterial richness | 3 | 3 | 2282.333 | 2256.000 | 29.023 | 8.185 | 0.985 | 0.748 |
| T5-T1 | Bacterial richness | 3 | 3 | 2253.000 | 2256.000 | 66.686 | 8.185 | -0.050 | 0.667 |
| T2-T1 | Bacterial shannon | 3 | 3 | 6.630 | 6.629 | 0.130 | 0.190 | 0.005 | 0.667 |
| T3-T1 | Bacterial shannon | 3 | 3 | 6.666 | 6.629 | 0.065 | 0.190 | 0.207 | 0.670 |
| T4-T1 | Bacterial shannon | 3 | 3 | 6.713 | 6.629 | 0.042 | 0.190 | 0.485 | 0.686 |
| T5-T1 | Bacterial shannon | 3 | 3 | 6.594 | 6.629 | 0.207 | 0.190 | -0.142 | 0.668 |
| T2-T1 | Bacterial evenness | 3 | 3 | 0.858 | 0.859 | 0.015 | 0.024 | -0.022 | 0.667 |
| T3-T1 | Bacterial evenness | 3 | 3 | 0.862 | 0.859 | 0.009 | 0.024 | 0.148 | 0.668 |
| T4-T1 | Bacterial evenness | 3 | 3 | 0.868 | 0.859 | 0.004 | 0.024 | 0.437 | 0.683 |
| T5-T1 | Bacterial evenness | 3 | 3 | 0.854 | 0.859 | 0.024 | 0.024 | -0.148 | 0.669 |
| T2-T1 | Fungal richness | 3 | 3 | 483.333 | 443.667 | 17.039 | 47.522 | 0.887 | 0.732 |
| T3-T1 | Fungal richness | 3 | 3 | 447.333 | 443.667 | 5.508 | 47.522 | 0.086 | 0.667 |
| T4-T1 | Fungal richness | 3 | 3 | 490.667 | 443.667 | 25.716 | 47.522 | 0.981 | 0.747 |
| T5-T1 | Fungal richness | 3 | 3 | 476.667 | 443.667 | 22.189 | 47.522 | 0.710 | 0.709 |
| T2-T1 | Fungal shannon | 3 | 3 | 4.446 | 4.096 | 0.202 | 0.518 | 0.711 | 0.709 |
| T3-T1 | Fungal shannon | 3 | 3 | 4.277 | 4.096 | 0.310 | 0.518 | 0.340 | 0.676 |
| T4-T1 | Fungal shannon | 3 | 3 | 4.464 | 4.096 | 0.066 | 0.518 | 0.797 | 0.720 |
| T5-T1 | Fungal shannon | 3 | 3 | 4.563 | 4.096 | 0.093 | 0.518 | 1.002 | 0.750 |
| T2-T1 | Fungal evenness | 3 | 3 | 0.719 | 0.672 | 0.029 | 0.075 | 0.673 | 0.704 |
| T3-T1 | Fungal evenness | 3 | 3 | 0.701 | 0.672 | 0.050 | 0.075 | 0.367 | 0.678 |
| T4-T1 | Fungal evenness | 3 | 3 | 0.721 | 0.672 | 0.016 | 0.075 | 0.727 | 0.711 |
| T5-T1 | Fungal evenness | 3 | 3 | 0.740 | 0.672 | 0.012 | 0.075 | 1.022 | 0.754 |
| T2-T1 | Acidobacteria | 3 | 3 | 0.243 | 0.251 | 0.042 | 0.079 | -0.098 | 0.667 |
| T3-T1 | Acidobacteria | 3 | 3 | 0.239 | 0.251 | 0.017 | 0.079 | -0.169 | 0.669 |
| T4-T1 | Acidobacteria | 3 | 3 | 0.233 | 0.251 | 0.035 | 0.079 | -0.236 | 0.671 |
| T5-T1 | Acidobacteria | 3 | 3 | 0.267 | 0.251 | 0.042 | 0.079 | 0.209 | 0.670 |
| T2-T1 | Actinobacteria | 3 | 3 | 0.072 | 0.062 | 0.004 | 0.005 | 1.875 | 0.960 |
| T3-T1 | Actinobacteria | 3 | 3 | 0.058 | 0.062 | 0.003 | 0.005 | -0.626 | 0.699 |
| T4-T1 | Actinobacteria | 3 | 3 | 0.072 | 0.062 | 0.008 | 0.005 | 1.181 | 0.783 |
| T5-T1 | Actinobacteria | 3 | 3 | 0.066 | 0.062 | 0.006 | 0.005 | 0.624 | 0.699 |
| T2-T1 | Armatimonadetes | 3 | 3 | 0.007 | 0.006 | 0.002 | 0.001 | 0.445 | 0.683 |
| T3-T1 | Armatimonadetes | 3 | 3 | 0.005 | 0.006 | 0.000 | 0.001 | -0.959 | 0.743 |
| T4-T1 | Armatimonadetes | 3 | 3 | 0.007 | 0.006 | 0.002 | 0.001 | 0.159 | 0.669 |
| T5-T1 | Armatimonadetes | 3 | 3 | 0.006 | 0.006 | 0.001 | 0.001 | -0.163 | 0.669 |
| T2-T1 | Bacteroidetes | 3 | 3 | 0.052 | 0.056 | 0.016 | 0.031 | -0.137 | 0.668 |
| T3-T1 | Bacteroidetes | 3 | 3 | 0.066 | 0.056 | 0.010 | 0.031 | 0.328 | 0.676 |
| T4-T1 | Bacteroidetes | 3 | 3 | 0.057 | 0.056 | 0.015 | 0.031 | 0.024 | 0.667 |
| T5-T1 | Bacteroidetes | 3 | 3 | 0.048 | 0.056 | 0.016 | 0.031 | -0.249 | 0.672 |
| T4-T1 | BD1.5 | 3 | 3 | 0.001 | 0.000 | 0.001 | 0.000 | 0.651 | 0.702 |
| T5-T1 | BD1.5 | 3 | 3 | 0.000 | 0.000 | 0.000 | 0.000 | 1.046 | 0.758 |
| T2-T1 | Candidate_division_OD1 | 3 | 3 | 0.004 | 0.003 | 0.001 | 0.001 | 0.733 | 0.711 |
| T3-T1 | Candidate_division_OD1 | 3 | 3 | 0.005 | 0.003 | 0.002 | 0.001 | 1.320 | 0.812 |
| T4-T1 | Candidate_division_OD1 | 3 | 3 | 0.003 | 0.003 | 0.000 | 0.001 | 0.412 | 0.681 |
| T5-T1 | Candidate_division_OD1 | 3 | 3 | 0.004 | 0.003 | 0.000 | 0.001 | 0.961 | 0.744 |
| T2-T1 | Candidate_division_OP11 | 3 | 3 | 0.000 | 0.000 | 0.000 | 0.000 | 0.865 | 0.729 |
| T3-T1 | Candidate_division_OP11 | 3 | 3 | 0.000 | 0.000 | 0.000 | 0.000 | 1.060 | 0.760 |
| T4-T1 | Candidate_division_OP11 | 3 | 3 | 0.000 | 0.000 | 0.000 | 0.000 | 1.384 | 0.826 |
| T5-T1 | Candidate_division_OP11 | 3 | 3 | 0.000 | 0.000 | 0.000 | 0.000 | 2.072 | 1.024 |
| T2-T1 | Candidate_division_OP3 | 3 | 3 | 0.000 | 0.000 | 0.000 | 0.000 | 0.074 | 0.667 |
| T3-T1 | Candidate_division_OP3 | 3 | 3 | 0.000 | 0.000 | 0.000 | 0.000 | 0.183 | 0.669 |
| T4-T1 | Candidate_division_OP3 | 3 | 3 | 0.000 | 0.000 | 0.000 | 0.000 | 0.282 | 0.673 |
| T5-T1 | Candidate_division_OP3 | 3 | 3 | 0.000 | 0.000 | 0.000 | 0.000 | 0.108 | 0.668 |
| T2-T1 | Candidate_division_TM7 | 3 | 3 | 0.006 | 0.004 | 0.002 | 0.001 | 1.386 | 0.827 |
| T3-T1 | Candidate_division_TM7 | 3 | 3 | 0.005 | 0.004 | 0.000 | 0.001 | 1.627 | 0.887 |
| T4-T1 | Candidate_division_TM7 | 3 | 3 | 0.005 | 0.004 | 0.000 | 0.001 | 1.906 | 0.970 |
| T5-T1 | Candidate_division_TM7 | 3 | 3 | 0.004 | 0.004 | 0.001 | 0.001 | 0.274 | 0.673 |
| T2-T1 | Candidate_division_WS3 | 3 | 3 | 0.010 | 0.014 | 0.005 | 0.001 | -0.771 | 0.716 |
| T3-T1 | Candidate_division_WS3 | 3 | 3 | 0.012 | 0.014 | 0.005 | 0.001 | -0.367 | 0.678 |
| T4-T1 | Candidate_division_WS3 | 3 | 3 | 0.011 | 0.014 | 0.002 | 0.001 | -1.451 | 0.842 |
| T5-T1 | Candidate_division_WS3 | 3 | 3 | 0.010 | 0.014 | 0.003 | 0.001 | -1.195 | 0.786 |
| T2-T1 | Candidate_division_WS6 | 3 | 3 | 0.000 | 0.000 | 0.000 | 0.000 | -0.690 | 0.706 |
| T3-T1 | Candidate_division_WS6 | 3 | 3 | 0.000 | 0.000 | 0.000 | 0.000 | 0.654 | 0.702 |
| T4-T1 | Candidate_division_WS6 | 3 | 3 | 0.000 | 0.000 | 0.000 | 0.000 | -0.513 | 0.689 |
| T5-T1 | Candidate_division_WS6 | 3 | 3 | 0.000 | 0.000 | 0.000 | 0.000 | -0.279 | 0.673 |
| T2-T1 | Chlamydiae | 3 | 3 | 0.002 | 0.002 | 0.000 | 0.001 | -0.831 | 0.724 |
| T3-T1 | Chlamydiae | 3 | 3 | 0.001 | 0.002 | 0.000 | 0.001 | -1.340 | 0.816 |
| T4-T1 | Chlamydiae | 3 | 3 | 0.001 | 0.002 | 0.000 | 0.001 | -0.976 | 0.746 |
| T5-T1 | Chlamydiae | 3 | 3 | 0.002 | 0.002 | 0.000 | 0.001 | -0.865 | 0.729 |
| T2-T1 | Chlorobi | 3 | 3 | 0.004 | 0.003 | 0.002 | 0.001 | 0.242 | 0.672 |
| T3-T1 | Chlorobi | 3 | 3 | 0.003 | 0.003 | 0.001 | 0.001 | -0.205 | 0.670 |
| T4-T1 | Chlorobi | 3 | 3 | 0.002 | 0.003 | 0.001 | 0.001 | -0.912 | 0.736 |
| T5-T1 | Chlorobi | 3 | 3 | 0.003 | 0.003 | 0.000 | 0.001 | -0.120 | 0.668 |
| T2-T1 | Chloroflexi | 3 | 3 | 0.057 | 0.052 | 0.003 | 0.011 | 0.427 | 0.682 |
| T3-T1 | Chloroflexi | 3 | 3 | 0.054 | 0.052 | 0.002 | 0.011 | 0.161 | 0.669 |
| T4-T1 | Chloroflexi | 3 | 3 | 0.058 | 0.052 | 0.003 | 0.011 | 0.605 | 0.697 |
| T5-T1 | Chloroflexi | 3 | 3 | 0.055 | 0.052 | 0.011 | 0.011 | 0.219 | 0.671 |
| T2-T1 | Cyanobacteria | 3 | 3 | 0.006 | 0.005 | 0.003 | 0.002 | 0.471 | 0.685 |
| T3-T1 | Cyanobacteria | 3 | 3 | 0.003 | 0.005 | 0.001 | 0.002 | -0.657 | 0.703 |
| T4-T1 | Cyanobacteria | 3 | 3 | 0.005 | 0.005 | 0.001 | 0.002 | 0.385 | 0.679 |
| T5-T1 | Cyanobacteria | 3 | 3 | 0.004 | 0.005 | 0.001 | 0.002 | -0.509 | 0.688 |
| T2-T1 | Deinococcus.Thermus | 3 | 3 | 0.000 | 0.000 | 0.000 | 0.000 | -0.591 | 0.696 |
| T3-T1 | Deinococcus.Thermus | 3 | 3 | 0.000 | 0.000 | 0.000 | 0.000 | -0.631 | 0.700 |
| T4-T1 | Deinococcus.Thermus | 3 | 3 | 0.000 | 0.000 | 0.000 | 0.000 | -0.570 | 0.694 |
| T5-T1 | Deinococcus.Thermus | 3 | 3 | 0.000 | 0.000 | 0.000 | 0.000 | -0.601 | 0.697 |
| T2-T1 | Elusimicrobia | 3 | 3 | 0.002 | 0.003 | 0.001 | 0.001 | -0.286 | 0.673 |
| T3-T1 | Elusimicrobia | 3 | 3 | 0.002 | 0.003 | 0.000 | 0.001 | -0.493 | 0.687 |
| T4-T1 | Elusimicrobia | 3 | 3 | 0.003 | 0.003 | 0.000 | 0.001 | -0.164 | 0.669 |
| T5-T1 | Elusimicrobia | 3 | 3 | 0.003 | 0.003 | 0.001 | 0.001 | -0.102 | 0.668 |
| T2-T1 | Fibrobacteres | 3 | 3 | 0.001 | 0.001 | 0.000 | 0.000 | 0.090 | 0.667 |
| T3-T1 | Fibrobacteres | 3 | 3 | 0.002 | 0.001 | 0.000 | 0.000 | 1.598 | 0.880 |
| T4-T1 | Fibrobacteres | 3 | 3 | 0.001 | 0.001 | 0.000 | 0.000 | -0.175 | 0.669 |
| T5-T1 | Fibrobacteres | 3 | 3 | 0.001 | 0.001 | 0.000 | 0.000 | -0.057 | 0.667 |
| T2-T1 | Firmicutes | 3 | 3 | 0.005 | 0.005 | 0.002 | 0.001 | 0.333 | 0.676 |
| T3-T1 | Firmicutes | 3 | 3 | 0.005 | 0.005 | 0.001 | 0.001 | 0.240 | 0.671 |
| T4-T1 | Firmicutes | 3 | 3 | 0.007 | 0.005 | 0.001 | 0.001 | 1.547 | 0.866 |
| T5-T1 | Firmicutes | 3 | 3 | 0.014 | 0.005 | 0.014 | 0.001 | 0.678 | 0.705 |
| T2-T1 | Gemmatimonadetes | 3 | 3 | 0.058 | 0.066 | 0.004 | 0.009 | -0.855 | 0.728 |
| T3-T1 | Gemmatimonadetes | 3 | 3 | 0.062 | 0.066 | 0.002 | 0.009 | -0.480 | 0.686 |
| T4-T1 | Gemmatimonadetes | 3 | 3 | 0.062 | 0.066 | 0.004 | 0.009 | -0.434 | 0.682 |
| T5-T1 | Gemmatimonadetes | 3 | 3 | 0.057 | 0.066 | 0.011 | 0.009 | -0.646 | 0.701 |
| T2-T1 | Nitrospirae | 3 | 3 | 0.013 | 0.016 | 0.004 | 0.003 | -0.548 | 0.692 |
| T3-T1 | Nitrospirae | 3 | 3 | 0.012 | 0.016 | 0.002 | 0.003 | -1.211 | 0.789 |
| T4-T1 | Nitrospirae | 3 | 3 | 0.017 | 0.016 | 0.003 | 0.003 | 0.299 | 0.674 |
| T5-T1 | Nitrospirae | 3 | 3 | 0.014 | 0.016 | 0.001 | 0.003 | -0.510 | 0.688 |
| T2-T1 | Planctomycetes | 3 | 3 | 0.015 | 0.015 | 0.003 | 0.004 | 0.137 | 0.668 |
| T3-T1 | Planctomycetes | 3 | 3 | 0.016 | 0.015 | 0.003 | 0.004 | 0.293 | 0.674 |
| T4-T1 | Planctomycetes | 3 | 3 | 0.015 | 0.015 | 0.002 | 0.004 | 0.149 | 0.669 |
| T5-T1 | Planctomycetes | 3 | 3 | 0.016 | 0.015 | 0.002 | 0.004 | 0.187 | 0.670 |
| T2-T1 | Proteobacteria | 3 | 3 | 0.394 | 0.391 | 0.030 | 0.056 | 0.063 | 0.667 |
| T3-T1 | Proteobacteria | 3 | 3 | 0.403 | 0.391 | 0.006 | 0.056 | 0.238 | 0.671 |
| T4-T1 | Proteobacteria | 3 | 3 | 0.396 | 0.391 | 0.028 | 0.056 | 0.090 | 0.667 |
| T5-T1 | Proteobacteria | 3 | 3 | 0.381 | 0.391 | 0.013 | 0.056 | -0.182 | 0.669 |
| T2-T1 | SHA.109 | 3 | 3 | 0.000 | 0.000 | 0.000 | 0.000 | -0.405 | 0.680 |
| T3-T1 | SHA.109 | 3 | 3 | 0.000 | 0.000 | 0.000 | 0.000 | 0.708 | 0.708 |
| T4-T1 | SHA.109 | 3 | 3 | 0.000 | 0.000 | 0.000 | 0.000 | 0.569 | 0.694 |
| T5-T1 | SHA.109 | 3 | 3 | 0.000 | 0.000 | 0.000 | 0.000 | -0.394 | 0.680 |
| T2-T1 | SM2F11 | 3 | 3 | 0.001 | 0.001 | 0.000 | 0.000 | -0.677 | 0.705 |
| T3-T1 | SM2F11 | 3 | 3 | 0.001 | 0.001 | 0.000 | 0.000 | -1.581 | 0.875 |
| T4-T1 | SM2F11 | 3 | 3 | 0.001 | 0.001 | 0.000 | 0.000 | -0.574 | 0.694 |
| T5-T1 | SM2F11 | 3 | 3 | 0.001 | 0.001 | 0.000 | 0.000 | 0.206 | 0.670 |
| T2-T1 | Spirochaetae | 3 | 3 | 0.000 | 0.001 | 0.000 | 0.000 | -0.613 | 0.698 |
| T3-T1 | Spirochaetae | 3 | 3 | 0.001 | 0.001 | 0.000 | 0.000 | 0.699 | 0.707 |
| T4-T1 | Spirochaetae | 3 | 3 | 0.001 | 0.001 | 0.000 | 0.000 | 0.359 | 0.677 |
| T5-T1 | Spirochaetae | 3 | 3 | 0.000 | 0.001 | 0.000 | 0.000 | -3.008 | 1.421 |
| T2-T1 | Thermotogae | 3 | 3 | 0.001 | 0.001 | 0.001 | 0.001 | 0.090 | 0.667 |
| T3-T1 | Thermotogae | 3 | 3 | 0.001 | 0.001 | 0.001 | 0.001 | -0.292 | 0.674 |
| T4-T1 | Thermotogae | 3 | 3 | 0.001 | 0.001 | 0.000 | 0.001 | -0.095 | 0.667 |
| T5-T1 | Thermotogae | 3 | 3 | 0.001 | 0.001 | 0.000 | 0.001 | -0.031 | 0.667 |
| T2-T1 | TM6 | 3 | 3 | 0.001 | 0.001 | 0.000 | 0.000 | 0.328 | 0.676 |
| T3-T1 | TM6 | 3 | 3 | 0.001 | 0.001 | 0.000 | 0.000 | 0.032 | 0.667 |
| T4-T1 | TM6 | 3 | 3 | 0.000 | 0.001 | 0.000 | 0.000 | -0.462 | 0.684 |
| T5-T1 | TM6 | 3 | 3 | 0.001 | 0.001 | 0.000 | 0.000 | 0.118 | 0.668 |
| T2-T1 | Verrucomicrobia | 3 | 3 | 0.037 | 0.038 | 0.003 | 0.002 | -0.152 | 0.669 |
| T3-T1 | Verrucomicrobia | 3 | 3 | 0.037 | 0.038 | 0.003 | 0.002 | -0.232 | 0.671 |
| T4-T1 | Verrucomicrobia | 3 | 3 | 0.035 | 0.038 | 0.001 | 0.002 | -1.754 | 0.923 |
| T5-T1 | Verrucomicrobia | 3 | 3 | 0.034 | 0.038 | 0.005 | 0.002 | -0.866 | 0.729 |
| T2-T1 | WCHB1.60 | 3 | 3 | 0.001 | 0.001 | 0.000 | 0.000 | 0.721 | 0.710 |
| T3-T1 | WCHB1.60 | 3 | 3 | 0.001 | 0.001 | 0.000 | 0.000 | 0.707 | 0.708 |
| T4-T1 | WCHB1.60 | 3 | 3 | 0.001 | 0.001 | 0.000 | 0.000 | -0.310 | 0.675 |
| T5-T1 | WCHB1.60 | 3 | 3 | 0.001 | 0.001 | 0.000 | 0.000 | 0.154 | 0.669 |
| T2-T1 | WD272 | 3 | 3 | 0.006 | 0.004 | 0.002 | 0.003 | 0.720 | 0.710 |
| T3-T1 | WD272 | 3 | 3 | 0.005 | 0.004 | 0.001 | 0.003 | 0.197 | 0.670 |
| T4-T1 | WD272 | 3 | 3 | 0.005 | 0.004 | 0.002 | 0.003 | 0.331 | 0.676 |
| T5-T1 | WD272 | 3 | 3 | 0.006 | 0.004 | 0.004 | 0.003 | 0.520 | 0.689 |
| T2-T1 | Ascomycota | 3 | 3 | 0.698 | 0.683 | 0.010 | 0.125 | 0.135 | 0.668 |
| T3-T1 | Ascomycota | 3 | 3 | 0.647 | 0.683 | 0.042 | 0.125 | -0.309 | 0.675 |
| T4-T1 | Ascomycota | 3 | 3 | 0.640 | 0.683 | 0.048 | 0.125 | -0.367 | 0.678 |
| T5-T1 | Ascomycota | 3 | 3 | 0.684 | 0.683 | 0.087 | 0.125 | 0.007 | 0.667 |
| T2-T1 | Basidiomycota | 3 | 3 | 0.014 | 0.032 | 0.003 | 0.008 | -2.527 | 1.199 |
| T3-T1 | Basidiomycota | 3 | 3 | 0.020 | 0.032 | 0.015 | 0.008 | -0.823 | 0.723 |
| T4-T1 | Basidiomycota | 3 | 3 | 0.013 | 0.032 | 0.005 | 0.008 | -2.480 | 1.179 |
| T5-T1 | Basidiomycota | 3 | 3 | 0.016 | 0.032 | 0.003 | 0.008 | -2.209 | 1.073 |
| T2-T1 | Chytridiomycota | 3 | 3 | 0.012 | 0.004 | 0.010 | 0.002 | 0.913 | 0.736 |
| T3-T1 | Chytridiomycota | 3 | 3 | 0.012 | 0.004 | 0.009 | 0.002 | 0.956 | 0.743 |
| T4-T1 | Chytridiomycota | 3 | 3 | 0.008 | 0.004 | 0.002 | 0.002 | 1.556 | 0.869 |
| T5-T1 | Chytridiomycota | 3 | 3 | 0.013 | 0.004 | 0.012 | 0.002 | 0.857 | 0.728 |
| T2-T1 | Glomeromycota | 3 | 3 | 0.028 | 0.030 | 0.021 | 0.010 | -0.088 | 0.667 |
| T3-T1 | Glomeromycota | 3 | 3 | 0.071 | 0.030 | 0.034 | 0.010 | 1.290 | 0.805 |
| T4-T1 | Glomeromycota | 3 | 3 | 0.036 | 0.030 | 0.025 | 0.010 | 0.226 | 0.671 |
| T5-T1 | Glomeromycota | 3 | 3 | 0.022 | 0.030 | 0.028 | 0.010 | -0.323 | 0.675 |
| T2-T1 | Zygomycota | 3 | 3 | 0.033 | 0.037 | 0.017 | 0.014 | -0.189 | 0.670 |
| T3-T1 | Zygomycota | 3 | 3 | 0.038 | 0.037 | 0.019 | 0.014 | 0.057 | 0.667 |
| T4-T1 | Zygomycota | 3 | 3 | 0.049 | 0.037 | 0.010 | 0.014 | 0.816 | 0.722 |
| T5-T1 | Zygomycota | 3 | 3 | 0.043 | 0.037 | 0.012 | 0.014 | 0.365 | 0.678 |

**Supplementary Table 1**. The effect size of physicochemical properties, nutrients, crop nutrients, microbial biomass carbon and nitrogen, enzyme activities, bacteria and fungus diversity, bacteria and fungus phylum between monocropping and intercropping.
